# Mice with an autism‐associated R451C mutation in neuroligin‐3 show a cautious but accurate response style in touchscreen attention tasks

**DOI:** 10.1111/gbb.12757

**Published:** 2021-07-02

**Authors:** Emma L. Burrows, Carlos May, Thomas Hill, Leonid Churliov, Katherine A. Johnson, Anthony J. Hannan

**Affiliations:** ^1^ Florey Institute of Neuroscience and Mental Health, University of Melbourne Parkville Victoria Australia; ^2^ Florey Institute of Neuroscience and Mental Health Heidelberg Victoria Australia; ^3^ School of Psychological Sciences University of Melbourne Parkville Victoria Australia; ^4^ Department of Anatomy and Neuroscience University of Melbourne Parkville Victoria Australia

**Keywords:** 5‐choice serial reaction task, attention, autism spectrum disorder, continuous performance task, gene‐edited mouse model, information processing, neuroligin‐3, R451C mutation, response times, touchscreen tests

## Abstract

One of the earliest identifiable features of autism spectrum disorder (ASD) is altered attention. Mice expressing the ASD‐associated R451C mutation in synaptic adhesion protein neuroligin‐3 (NL3) exhibit impaired reciprocal social interactions and repetitive and restrictive behaviours. The role of this mutation in attentional abnormalities has not been established. We assessed attention in male NL3^R451C^ mice using two well‐established tasks in touchscreen chambers. In the 5‐choice serial reaction task, rodents were trained to attend to light stimuli that appear in any one of five locations. While no differences between NL3^R451C^ and WT mice were seen in accuracy or omissions, slower response times and quicker reward collection latencies were seen across all training and probe trials. In the rodent continuous‐performance test, animals were required to discriminate, and identify a visual target pattern over multiple distractor stimuli. NL3^R451C^ mice displayed enhanced ability to attend to stimuli when task‐load was low during training and baseline but lost this advantage when difficulty was increased by altering task parameters in probe trials. NL3^R451C^ mice made less responses to the distractor stimuli, exhibiting lower false alarm rates during all training stages and in probe trials. Slower response times and quicker reward latencies were consistently seen in NL3^R451C^ mice in the rCPT. Slower response times are a major cognitive phenotype reported in ASD patients and are indicative of slower processing speed. Enhanced attention has been shown in a subset of ASD patients and we have demonstrated this phenotype also exists in the NL3^R451C^ mouse model.

## Graphical abstract

NL3 mice displayed enhanced ability to attend to stimuli when task‐load was low but lost this advantage when difficulty was high.

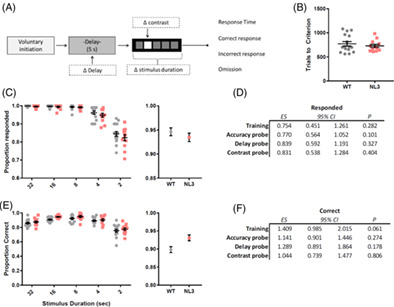

## 1 INTRODUCTION

Attentional abnormalities in autism spectrum disorder (ASD) are well documented.^1^ Estimates of co‐morbidity with attention‐deficit hyperactivity disorder (ADHD) range from 41% to 78% in individuals with ASD.^2^, ^3^, ^4^ As one of the earliest identifiable features of the condition,^5^, ^6^ attention has become the focus of a growing body of research that highlights the numerous changes to such cognitive processes. Nevertheless, results in this area of research are diverse and often conflicting. Although some studies report an impairment in selective attention and demonstrate increased levels of distractibility,^7^ other studies point to an enhanced ability.^8^ This discrepancy can be resolved by scrutinising attentional processing and the underlying neurobiology in animal models.

Due to the genetic heterogeneity of ASD it is not straightforward to model the disorder in animals. However, among many genes that have been shown to contribute to the manifestation of ASD a select few that are highly penetrant and are implicated in synaptic transmission, a process thought central to the pathology of ASD. These highly penetrant synaptic mutations can be introduced to mice and behavioural phenotypes recapitulating ASD‐like traits can then be interrogated. One mouse model contains a point mutation encoding an arginine‐to‐cysteine residue (R451C) substitution in neuroligin‐3 (NL3), identified in two Swedish brothers with ASD.^9^ NL3 is a synaptic cell‐adhesion protein that mediates trans‐synaptic signalling with presynaptic binding partner neurexins, and shapes neural network properties by recruiting and maintaining postsynaptic machinery.^10^ The R451C mutation is located on the X‐chromosome, causes a 90% reduction in NL3 protein at the cell membrane^11^ and, when modelled in mice, impairs synaptic properties and disrupts neural networks without completely abolishing synaptic transmission.^11^, ^12^, ^13^, ^14^, ^15^ Although this specific mutation is rare in clinical populations, a clustering of ASD associated mutations found in neurexins, neuroligins and their downstream binding partner Shank3^9^, ^16^, ^17^, ^18^ add to the hypothesis that disruption of this pathway contributes to the disorder. Our group, and others, have discovered impairments in NL3^R451C^ mice in core behavioural traits, including social impairment, presence of restrictive and repetitive behaviours and aggression;^11^, ^12^, ^13^, ^19^, ^20^, ^21^, ^22^, ^23^, ^24^ however, the role of this mutation in attentional processing has not been previously investigated.

Despite the significance of attention as an essential prerequisite for a variety of cognitive functions, limited assays for testing attention in rodents have been available. Touchscreen technology allows for a direct comparison between mouse and human behaviour.^25^ Recently published findings demonstrate that the cognitive functions measured by these touchscreens in mice are directly comparable to human cognition measured using touchscreen neuropsychological batteries (e.g., CANTAB) in clinical populations.^26^ In earlier stages of training, mice learn to discriminate between visual stimuli projected onto a touch‐sensitive computer screen and tasks can then be scaled in complexity to mimic stimuli (objects and locations on a computer screen), and responses (screen touches) used in human cognitive tests. Recently, touchscreen‐based attention tests for use in rodents have been adapted directly from clinical tasks, conferring greater translatability to scrutinise cognitive processes in genetic mouse models.

We assessed attention in male NL3^R451C^ mice using well‐established rodent tasks, the 5‐choice serial reaction task (5‐CSRTT) and the recently developed rodent continuous performance test (rCPT).^27^, ^28^ The 5‐CSRTT is a well characterised rodent test of visuospatial attention where animals are trained to attend to stimuli that appear in any one of five locations after a short delay.^28^ While commonly employed using operant chambers,^28^ it has been adapted for use in touchscreens.^29^ Optimal performance in this task requires mice to attend to the entirety of the touchscreen in order to detect a short flash of light. The 5‐CSRTT is experimenter driven, as rodents are required to initiate the next trial after collecting a reward and long periods where rodents are not engaged in the task can arise. In clinical studies, attentional paradigms are apparatus driven to ensure the patient must be actively engaged in the task.^30^ The rCPT was implemented to remove the visuospatial and experimenter driven elements of the 5‐CSRTT to more comprehensively probe attention in the NL3 model. The rCPT has been developed from the human continuous performance task (CPT), is a measure of sustained and selective attention^27^ and consists of responding only to a single target stimulus while inhibiting responses to distracter or “noise” stimuli. All images are successively displayed in a single, central location in the touchscreen. Although the 5CSRTT and the rCPT measure overlapping constructs of attention,^28^ there exist some important differences. The 5‐CSRTT paradigm includes spatial unpredictability, which is intended to assess divided spatially attention (the allocation of limited processing resources to broad areas of sensory space, not to be confused with performing two tasks simultaneously). The rCPT differs in that it requires discrimination of a target image against non‐target images. This increase in difficulty has been considered essential to properly tax vigilance.^31^ By using both these paradigms complimentarily, this study aims to provide a complete picture of attentional function in the NL3^R451C^ mouse.

## 2 METHODS

### 2.1 Animal husbandry and food‐restriction

C57/Bl6; 129‐Nlgn3tm1Sud/J mice, commercially purchased from Jackson Laboratories (Bar Harbour, Maine USA) were backcrossed with the C57BL6 strain for more than F10 generations. Male WT (Y/X) and NL3^R451C^ (Y/X‐NL3^R451C^) mice were generated by mating heterozygous females with WT males. At 2 weeks of age tail biopsies were taken for genotyping, which was performed commercially by Transnetyx (Cordova, Tennessee USA). Three cohorts of mice were bred in individually ventilated cages (IVC) and at 4 weeks of age mice and housed in open‐top cages in groups of 3–4, with unlimited access to food and water. For touchscreen experiments, two cohorts of mice were tested on separate tasks (5CSRTT and: WT = 14, NL3 = 11; rCPT: WT = 12, NL3 = 10; see timeline for both tasks, Supplementary Figure 1). Prior to testing, free feeding weight (FFW) was determined at 7 weeks of age and mice were phase shifted to reverse light cycle, which operated on a 12‐h light/dark cycle, commencing at 7 a.m. (dark phase), maintained at 22°C (+/− 1°C). All touchscreen testing occurred in the dark phase, and red light was used when handling was required. From 7 weeks of age mice were also singly housed due to a previously characterised aggression phenotype.^20^ Mice were gradually food restricted to 85% FFW from 8 weeks of age to ensure motivation to perform touchscreen tasks at 9 weeks of age. Seven‐week FFW was defined as the minimum healthy weight for the duration of testing. To allow normal development, FFW was increased by 1 g per week (based on normal C57Bl6 male weight gain, Jax® Body Weight information for C57Bl6) until mice were 13 weeks of age, which was then defined as the maximum healthy weight for the duration of testing. For saccharine preference test, mice (WT = 10; NL3 = 6) were singly housed, food restricted to 85% FFW (to match conditions of touchscreen testing) and assessed in their home‐cages. The Florey Institute of Neuroscience and Mental Health Ethics Committee approved all experimental protocols.

### 2.2 Saccharine preference

Mice were given access to both 0.1% saccharin and tap water for two overnight periods (from 6 p.m. to 9 a.m.). The location of the saccharin bottle (left or right) was changed for each mouse on the second period to avoid the development of place preference. Water and saccharin bottles were weighed before and after each period to determine the amount consumed. Percentage saccharin preference was derived by calculating the proportion of saccharin consumed relative to total fluid intake.

### 2.3 Touchscreen apparatus

Mice were tested using touchscreen operant chambers (Campden Instruments Ltd., UK), described previously.^32^ Testing was conducted in a trapezoidal‐shaped operant chamber composed of a metal floor, a reward delivery magazine, a touchscreen, two infrared (IR) beams for motor activity detection and black Perspex side‐walls. The chamber was housed inside a sound‐ and light‐attenuating box with a house light, a tone generator, a ventilating fan and an IR camera. ABET software provided by Campden Instruments controlled the system and collected data. Black Perspex masks were placed over the touchscreen with three response windows (each square 7 × 7 cm^2^; for CPT, PR, FR and extinction). Liquid reward (Nippy's Iced Strawberry Milk; Knispel Brothers Pty Ltd) was provided to motivate performance. The black Perspex mask, chamber floor and walls and the excrement tray were all cleaned with 80% ethanol between testing sessions.

### 2.4 Touchscreen pre‐training

Mice were trained through iterative steps to nose poke stimuli on the screen for a 7 μl liquid reward and then moved onto one of the two cognitive tasks outlined below. Mice were 9 weeks old at the onset of behavioural testing and testing was performed with two cohorts of male mice behavioural training began with a 20 min habituation session to the chamber. On subsequent days, mice were trained to associate a reward with a stimulus on the screen. A 3 kHz reward tone and illumination of the reward‐dispensing magazine, indicated the location for reward. Responses to the stimuli resulted in ×3 the reward, while no response resulted in ×1. Once mice collected the maximum number of rewards, they were required to touch the white square for reward. Mice were “punished” with a 5 s time‐out and illumination of the chamber for touching other areas of the screen while the image was displayed or prematurely touching the screen. Mice were individually weighed and fed immediately after their testing session and were consistently assessed in the same chamber to remove any potential confounding factors of a novel‐testing environment.

### 2.5 5‐choice serial reaction time task

In the 5‐CSRTT, mice were trained to respond, via nose poke, to brief stimuli (white square) presented pseudorandomly in one of five locations.^29^ During each trial, mice were required to voluntarily initiate by head poking the food well, which initiated a short delay to the stimulus presentation (default delay, 5 s). If an incorrect response was made (touches during delay or in hole where stimuli did not appear) or a missed trial occurred (not responding during stimulus presentation or within the limited hold [LH] period of 5 s after stimulus presentation, kept consistent throughout training and probes), mice were punished by a 5 s timeout along with illumination of the main light. After a response was recorded, 10 s inter trial interval (ITI) followed, after which the food well would light up to indicate the next trial was ready to be initiated. Mice were trained to acquire the task using decreasing stimulus duration settings, (32, 16, 8, 4, and 2 s). Mice were trained to criterion for two consecutive days before advancing to the next stimulus duration stage. Each 5‐CSRTT session consisted of 50 trials and mice were required to respond to >80% of trials with >80% accuracy for two consecutive days to advance to the next stimulus duration stage. All mice were held at default stimulus duration (2 s) until the cohort had acquired the task before progressing to probe trials. Once each individual mouse had reached criteria of the final training stage, they were rested without daily training, while mice not at criteria continued their training. Mice on rest were given a reminder training session twice per week, except when they fell below criterion, in which case they were tested until criterion was reached again. Many aspects of their performance were automatically recorded (see below for more information) in order to detect differences in sustained, divided and spatial attention.

After the successful completion of 5‐CSRTT training, stimulus duration (accuracy), inter‐trial interval (delay) and stimulus brightness (contrast) were altered to probe different aspects of attention. All probes were administered in blocks of two consecutive sessions (2 × sessions = 100 trials) with at least two sessions of 2 s stimulus duration between these blocks to ensure stable performance. To test visual spatial attention (accuracy probe), mice were tested on five blocks of reduced stimulus durations (1, 0.8, 0.6, 0.4, and 0.2 s). Vigilance and response inhibition (delay probe) were tested by either increasing (5, 6, 7, and 8 s presented variably within session) or decreasing (2, 3, 4, and 5 s) the inter‐trial interval immediately before stimulus presentation. Mice were tested on two blocks of both each long and short delays. Stimulus brightness (contrast probe) was reduced (100%, 80%, 60%, 40%, and 20%, presented variably within session) to uncover visual deficits that could potentially influence attentional performance.

### 2.6 5‐choice data collection

Responses to each trial were recorded as premature, correct, incorrect, or omissions. Additional parameters were collected to allow more specificity in cognitive phenotyping. These included response latency, reward collection latency, perseverative responses, initiation latency and beam breaks per trial. For subsequent analyses, measures were therefore conceptualised in the following way: Premature/non‐premature, response/omission and correct/incorrect were treated as binary outcomes and hence were dummy coded to 1/0, respectively. Premature/non‐premature was analysed in all recorded trials, response/omission was only analysed for non‐premature trials and correct/incorrect was only analysed for trials where a response was recorded. Initiation latency, response latency and reward collection latency were treated as continuous variables. Initiation latency analysis was performed for all trials. Response Latency analysis was performed only when a response was recorded and reward collection latency analysis was done only when the response was correct. Beam break data were treated as counts.

### 2.7 Rodent‐continuous performance test rCPT training

The rCPT measures various aspects of attention by training mice to respond to a single reward coupled stimulus (S+) and inhibit their response to four distractor stimuli (S−) and has been described previously.^27^ Individual stimuli were sequentially presented in the same location, mice were required to respond selectively to S+, and attentional load was increased throughout training in four stages. Throughout testing, a LH period of 0.5 s was used and terminated when 100 rewards were collected or when 45 min had elapsed.

### 2.8 Stage 1: White square recognition

Initially animals were required to identify a white square image, which was presented for 10 s, and make a response within the LH period (10.5 s). A response within the LH period was recorded as a 'hit' and the mouse was rewarded. Failure to make a response in the LH period was recorded as a 'miss'. Mice progressed to the next stage when they reached criterion; collection of >60 rewards for two consecutive days. The white square image was no longer used in rCPT training after Stage 1.

### 2.9 Stage 2: S+ recognition

Mice were allocated either a horizontal or vertical lined image as their S+ stimulus, counterbalanced across genotype and session. S+ stimulus presentation lasted for 5 s; a response within the LH period (5.5 s) was recorded as a hit. Failure to respond was recorded as a miss. Mice were moved to the next stage when they reached criterion; collection of >60 rewards for two consecutive days.

### 2.10 Stage 3: Detection of one S+ and one S−

In Stage 3, mice were exposed to a snowflake distractor image (S−) as well as their allocated S+ image; the probability of S+ occurrence was 50%. Duration of stimulus presentation was reduced to 2 s with a LH period of 2.5 s. A response to S− initiated a 5 s time out period where the chamber 'house light' was turned on. This effectively served as a punishment period to notify the animal that they had made an incorrect selection. Completion of the time out period initiated a correction trial where the animal had another chance to learn to inhibit their response to the S−. Correctly inhibiting a response to the S− was recorded as a correct rejection. The criterion to progress to the next stage of training was a *d*' ≥0.60 for three consecutive days, with a minimum of 7 days of training. The snowflake image was no longer used in rCPT training.

### 2.11 Stage 4.1 and 4.2: Detection of one S+ and four S−

The attentional load was increased in Stage 4 by introducing four deterring S−. The probability of S+ occurrence was initially 50%, until stable group performance was reached, and then was reduced to 33% (Stage 4.2). All other parameters remained the same as Stage 3. Stable performance was defined as a consistent *d*', ≥0.60 for a minimum of three consecutive days (assessed by repeated measures analyses of variance [ANOVA]).

### 2.12 CPT probes

After the successful completion of rCPT training, the following stimulus parameters were manipulated to increase the attentional load: Duration (2.0, 1.0, 0.5, and 0.2 s), contrast (100%, 50%, 25%, 12.5%), inter‐trial interval (2, 5, and 10 s.). All stimulus parameters were presented variably within sessions and three consecutive sessions of each probe were run. For the stimulus duration probe, the LH remained fixed at 2.5 s for all stimulus durations. Probes were designed to measure performance at varying difficulty; therefore there was no specified criterion for each session. Two or three baseline sessions (Stage 4.2) were run in between each probe to restore stable performance.

### 2.13 rCPT data collection

Responses (or lack of) were recorded as 'hits', 'misses', 'mistakes' (false alarms), 'correct rejections'. Additionally, data was collected to control for motivation, motor deficits and non‐selective responding ('centre touches during the ITI', time in session, response latency and reward collection latency). An analysis method based on signal detection theory was used to determine a mouse's ability to identify signal from noise. Hit rate (HR = hits/[hits+misses]) and false alarm rate (FAR = mistakes/(mistakes+correct rejections) were calculated. Looking at HR or FAR alone does not always give the best indication of performance, as a high HR may be coupled with a high FAR indicating non‐selective or impulsive responding. By combining the two measures *d*' and *c* can be calculated to analyse the discriminability (*d*' = z(HR)–z(FAR)) and criterion respectively (*c* = (−z(HR)‐z(FAR))/2). Discriminability (*d*') refers to sensitivity in which rodents can differentiate between stimuli (i.e., a high *d*' indicates a high level of discriminability). Criterion refers to rodents' willingness to respond. It should be noted that a high c value indicated a conservative response strategy and a low c value indicated a liberal response strategy.

### 2.14 Statistical analysis

5CSRTT trials to criterion data were analysed with a Mann–Whitney U test. All other measures within this task were similarly non‐normally distributed and analysed at the level of each trial using generalised linear, latent, and mixed models (GLLAMM) with robust standard error estimation and individual animals treated as random effects to reflect clustered nature of all observations within each animal. Justification for this statistical approach has been previously described.^33^ GLLAMM were run with genotype, day, trial, timestamp, and, if required, the relevant probe variable (duration, delay, contrast) as independent variables, with the odds of any response, a correct response, a premature response and the expected number of perseverative/blank touches as outcome variables. The adjusted effect of independent variables on outcomes were estimated by random‐effects logistic regression (binary data) as odds ratios (aORs), random‐effects Poisson regression (count data) as incidence rate ratios (aIRRs), or by median regression (continuous data) as median value (coefficients) with clustered errors with the effect sizes reported together with respective 95% confidence intervals to indicate the estimates' precision and corresponding two‐tailed p‐values for the hypothesis of no effect.

Compound measures of CPT performance (HR,FAR,*d*', and *c*) were normally distributed and two‐way repeated measures ANOVAs were performed. Genotype and a secondary factor (i.e., duration, contrast, etc.) were analysed as repeated measures. If a genotype*factor interaction was observed, pairwise comparisons were assessed via Bonferroni post hoc analysis. All other measures collected in the CPT were not normally distributed. Median regressions were used to analyse response latency and collection latency, whereas random‐effects poisson regressions were used to analyse centre touches and blank touches. IBM SPSS 25.0 (IBM, Armonk, New York, US) and STATA v13IC (StataCorp, College Station, TX, USA) were used to run statistical analyses. The data that support the findings of this study are available from the corresponding author upon reasonable request.

## 3 RESULTS

### 3.1 NL3^R451C^ mice show subtle attentional alterations in 5CSRTT training and probes

**FIGURE 1:**
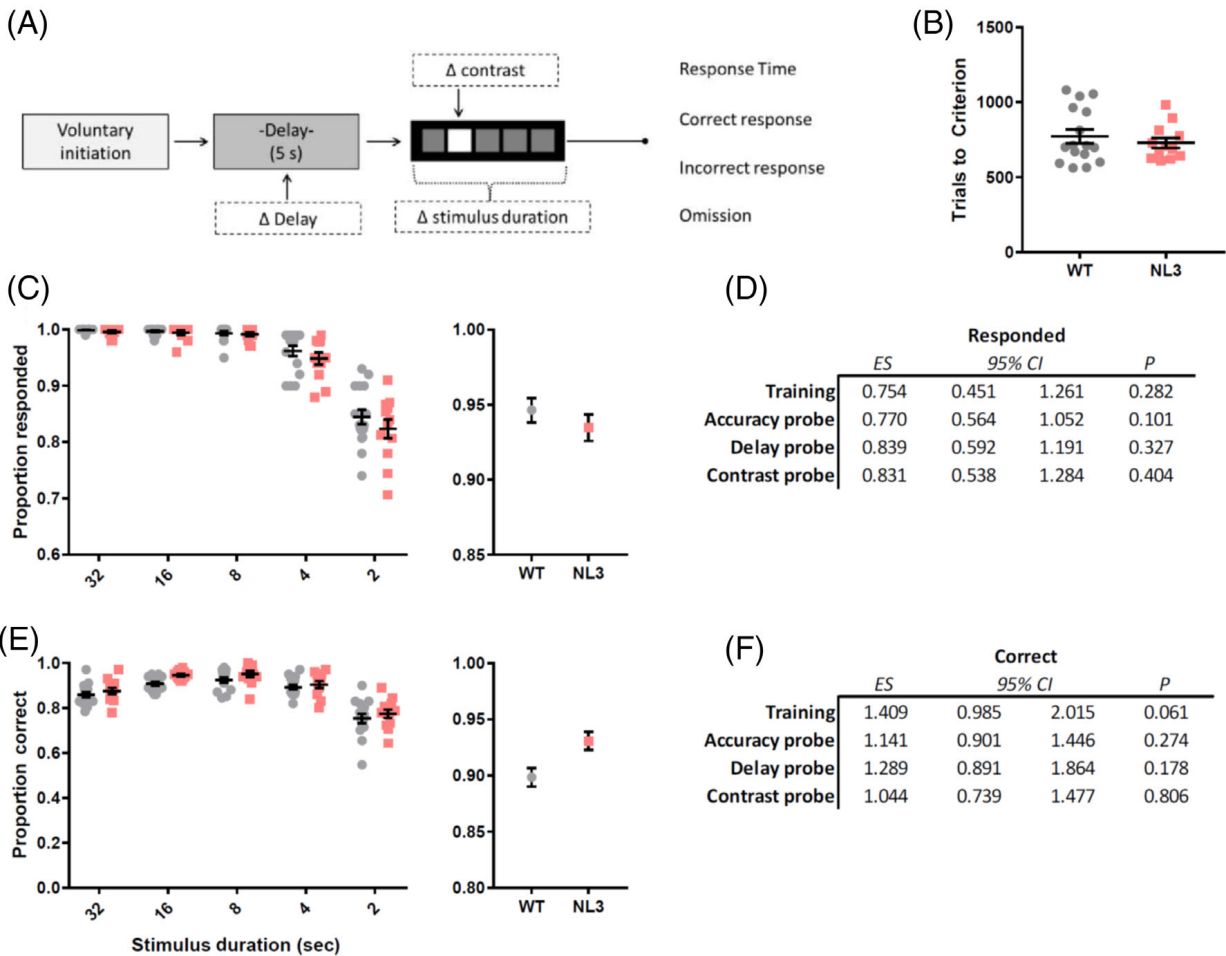
NL3^R451C^ mice successfully acquired the 5‐choice serial reaction task (5CSRTT). Schematic outlining task (A) NL3^R451C^ mice showed no gross differences in trials to criterion (B). Mice were trained over a number of reducing stimulus durations and both genotypes responded to a similar proportion of trials (C). This was consistent for accuracy, contrast and delay probes (D). No differences in accuracy between the groups were seen over the entire training period (E) or when mice were subjected to accuracy, delay and contrast probes (F). Data are presented as mean ± SEM for visualisation purposes

NL3^R451C^ mice showed no differences in 5CSRTT acquisition (Figure 1A), requiring similar numbers of trials to meet criterion as their WT littermates (Figure 1B; Mann–Whitney U, z = − 0.307 *p* = 0.759). Mice were trained on a number of reducing stimulus durations and both WT and NL3 ^R451C^ mice responded to a similar proportion of trials in all stages (Figure 1C; logistic regression, OR = 0.754, *p* = 0.282). When task parameters were manipulated to tax attention in mice, this similar likelihood of response was consistent for contrast (Figure 1D; logistic regression, OR = 0.831, *p* = 0.404), delay (logistic regression, OR = 0.839, *p* = 0.327) and accuracy probes (logistic regression, OR = 0.770, *p* = 0.101). NL3^R451C^ and WT mice showed comparable accuracy over the entire training period (Figure 1E; logistic regression, OR = 1.409, *p* = 0.061). Similarly, no genotype differences were seen when mice were subjected to accuracy (logistic regression, OR = 1.141, *p* = 0.274), contrast (logistic regression, OR = 1.044, *p* = 0.806), and delay (logistic regression, OR = 1.289, *p* = 0.178) probes. Increasing task difficulty during the probes led to the expected decrease in accuracy and increased omission rates in both genotypes; however, task difficulty did not impact genotypes differently (Supplementary Figure 2; Supplementary Table 1).

**FIGURE 2:**
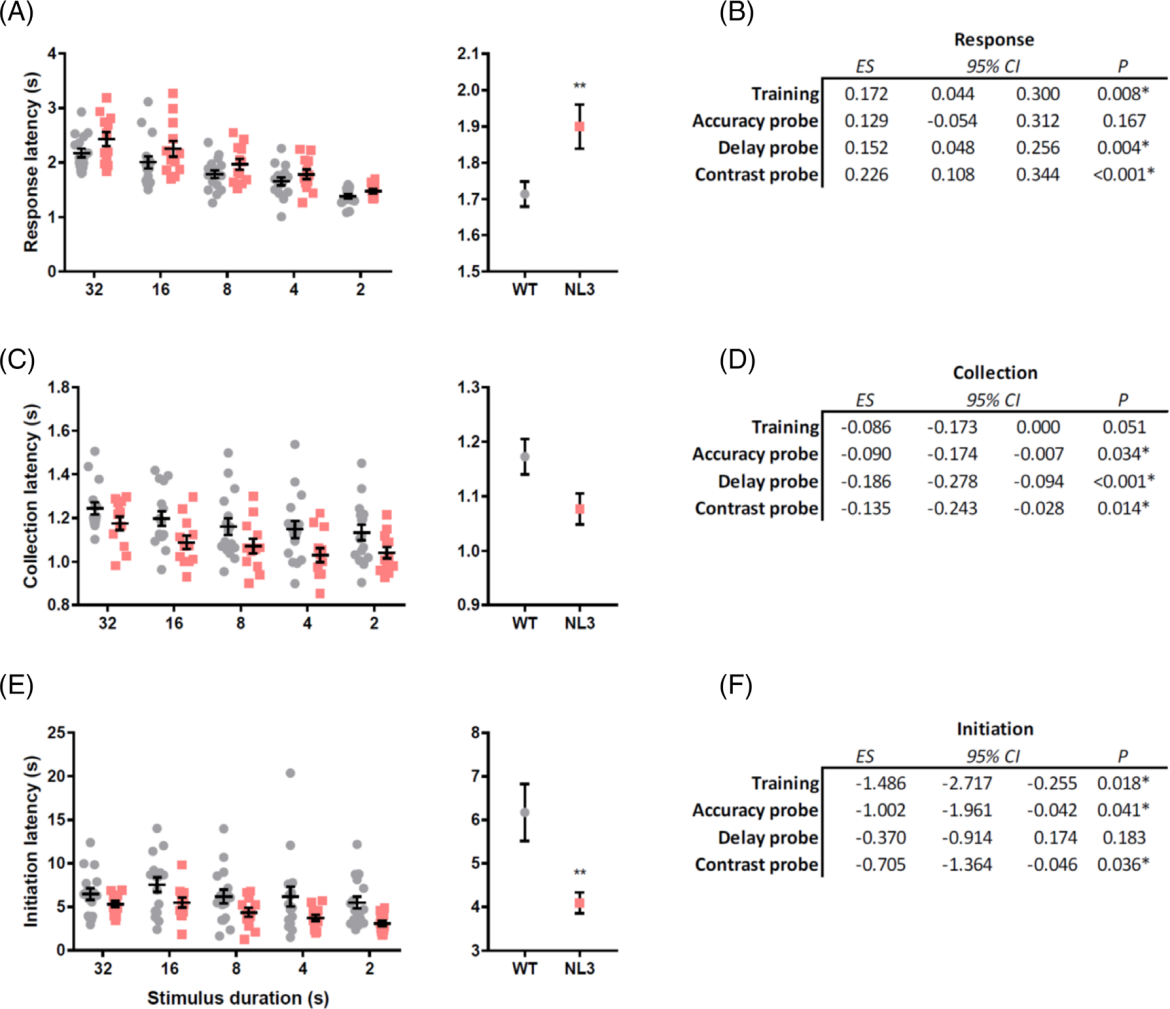
NL3^R451C^ mice exhibited slower response latencies, and quicker collection and initiation latencies across 5‐choice serial reaction task (5‐CSRTT) training and probes. NL3^R451C^ mice were slower to respond to stimuli during 5‐CSRTT training (A) and in delay and contrast but not accuracy probes (B). A trend for quicker reward collection latencies were seen in NL3^R451C^ mice over the training period (C). NL3 mice were quicker to collect rewards during all probes (D). NL3^R451C^ mice were also quicker to initiate trials in all training stages (E) and during the accuracy and contrast but not delay probes (F). Data are presented as mean ± SEM for visualisation purposes. *p < 0.05. **p < 0.01

NL3^R451C^ mice took longer to respond to stimuli throughout training (Figure 2A; median regression, Coef. = 0.172, *p* = 0.008). Longer response latencies in NL3^R451C^ mice were also seen in the delay (median regression, Coef. = 0.152, *p* = 0.004) and contrast (median regression, Coef. = 0.226, *p* < 0.001) probes. No differences in response latencies were seen in the accuracy probe (median regression, Coef. = 0.129, *p* = 0.167) and this was also consistent for all stimulus durations tested during this probe (genotype × duration interaction: Median regression, Coef. = −0.0135, *p* = 0.143). While slower response times may reflect lower motivation to perform the task, paradoxically NL3^R451C^ mice were quicker to collect rewards during the accuracy (Figure 2D; median regression, Coef. = − 0.090, *p* = 0.034), delay (median regression, Coef. = − 0.186, *p* < 0.001) and contrast (median regression, Coef. = − 0.135, *p* = 0.014) probes. This was also reflected as a non‐significant trend during the training period (Figure 2C; median regression, Coef. = − 0.086, *p* = 0.051). NL3^R451C^ mice were also quicker to initiate trials in all training stages (Figure 2E; median regression, Coef. = − 1.486, *p* = 0.018) and during accuracy (median regression, Coef. = − 1.002, *p* = 0.041) and contrast probes (median regression, Coef. = − 0.705, *p* = 0.036) but not the delay probe (median regression, Coef. = − 0.370, *p* = 0.183). Increasing task difficulty during the probes did not uniformly alter response latency, reward collection latency or initiation latency (For all 5‐CSRTT probe statistics and data refer to Supplementary Table 1 and Supplementary Figure 2, respectively).

**FIGURE 3:**
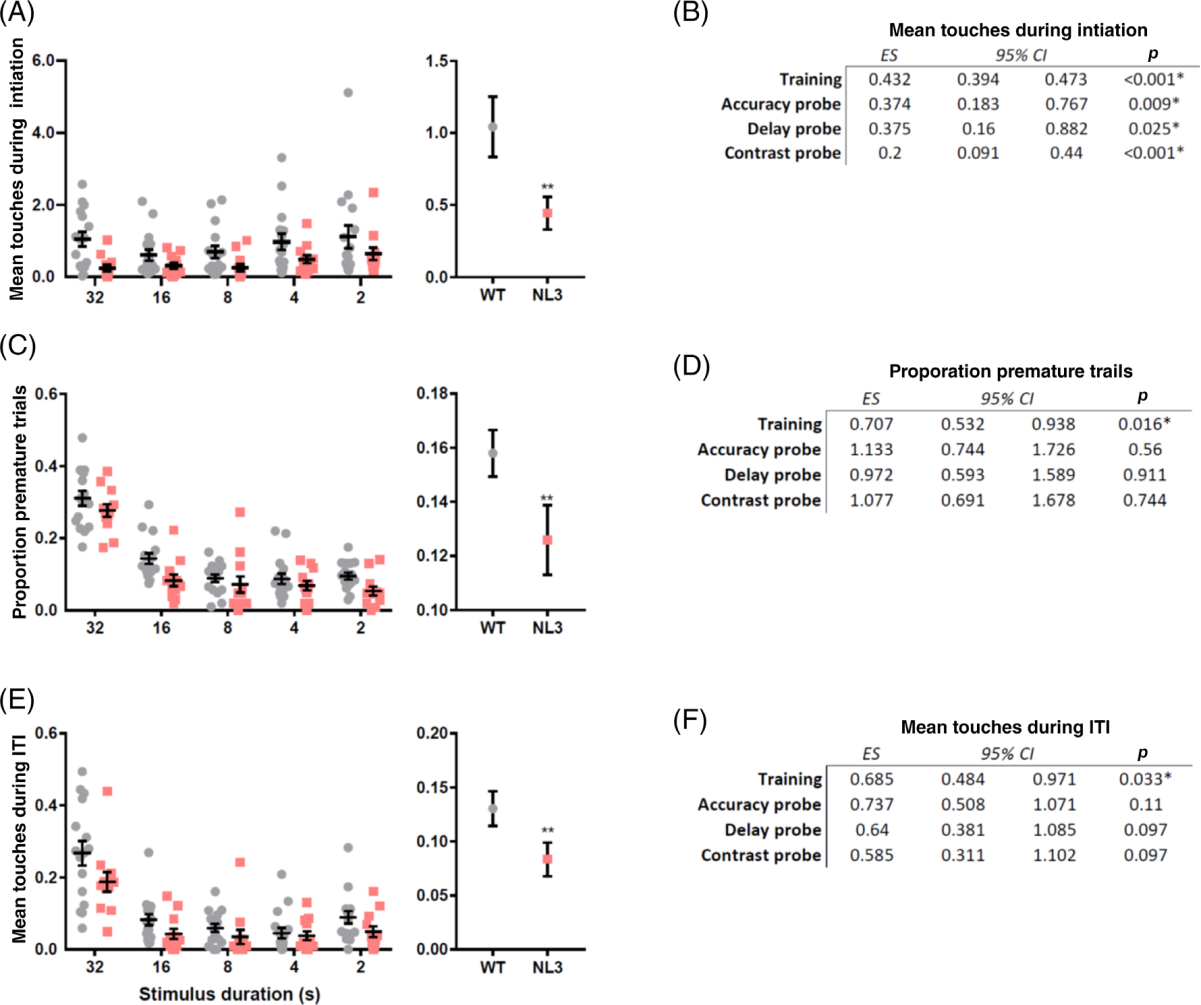
NL3^R451C^ mice showed less interest in the screen during 5‐choice serial reaction task (5‐CSRTT) training. NL3^R451C^ mice made less touches to the screen during the initiation period for 5‐CSRTT training (A) and all probes (B). NL3^R451C^ mice were also less likely to touch the screen prematurely following trial initiation for the training period (C) but not probes (D). During the 5 s inter trial interval (ITI), NL3^R451C^ mice also made fewer touches during training (E), but not probes (F). Data are presented as mean ± SEM for visualisation purposes. ** *p* < 0.01

NL3^R451C^ mice showed reduced interest in the screen during the initiation period, making less touches during this period for training (Figure 3A; Poisson regression, IRR = 0.432, *p* < 0.001). This was also the case for all probes (Figure 3B; accuracy: Poisson regression, IRR = 0.374, *p* = 0.009; delay: Poisson regression, IRR = 0.375, *p* = 0.025; contrast: Poisson regression, IRR = 0.200, *p* < 0.001). NL3^R451C^ mice were also less likely to touch the screen prematurely following trial initiation during the training period (training; Figure 3C: logistic regression, OR = 0.707, *p* = 0.016) but not during probes (Figure 3D; accuracy: Logistic regression, OR = 1.133, *p* = 0.56; delay: Logistic regression, OR = 0.972, *p* = 0.911; contrast: Logistic regression, OR = 1.077, *p* = 0.744). A similar pattern of disinterest in the screen was also seen during the 5 s ITI. NL3^R451C^ mice made fewer ITI touches to the screen during the training period (Figure 3E; Poisson regression, IRR = 0.685, *p* = 0.033) but not during the probes (Figure 3F; accuracy: Poisson regression, IRR = 0.737, *p* = 0.11; delay: Poisson regression, IRR = 0.640, *p* = 0.097; contrast: Poisson regression, IRR = 0.585, *p* = 0.097). NL3^R451C^ mice exhibited low interactions with the screen despite a modest increase in beam breaks at the front of the screen, potentially indicative of hyperactivity or different scanning strategies in this task (Supplementary Table 1). As expected, extending the stimulus delay during the delay probe led to increased premature responses. Aside from this, initiation touches, premature responses, and ITI touches remained relatively constant as task difficulty increased during the 5CSRTT probes (For all 5‐CSRTT probe statistics and data refer to Supplementary Table 1 and Supplementary Figure 2, respectively).

### 3.2 NL3^R451C^ mice show greater ability to respond to stimuli during rCPT training

**FIGURE 4:**
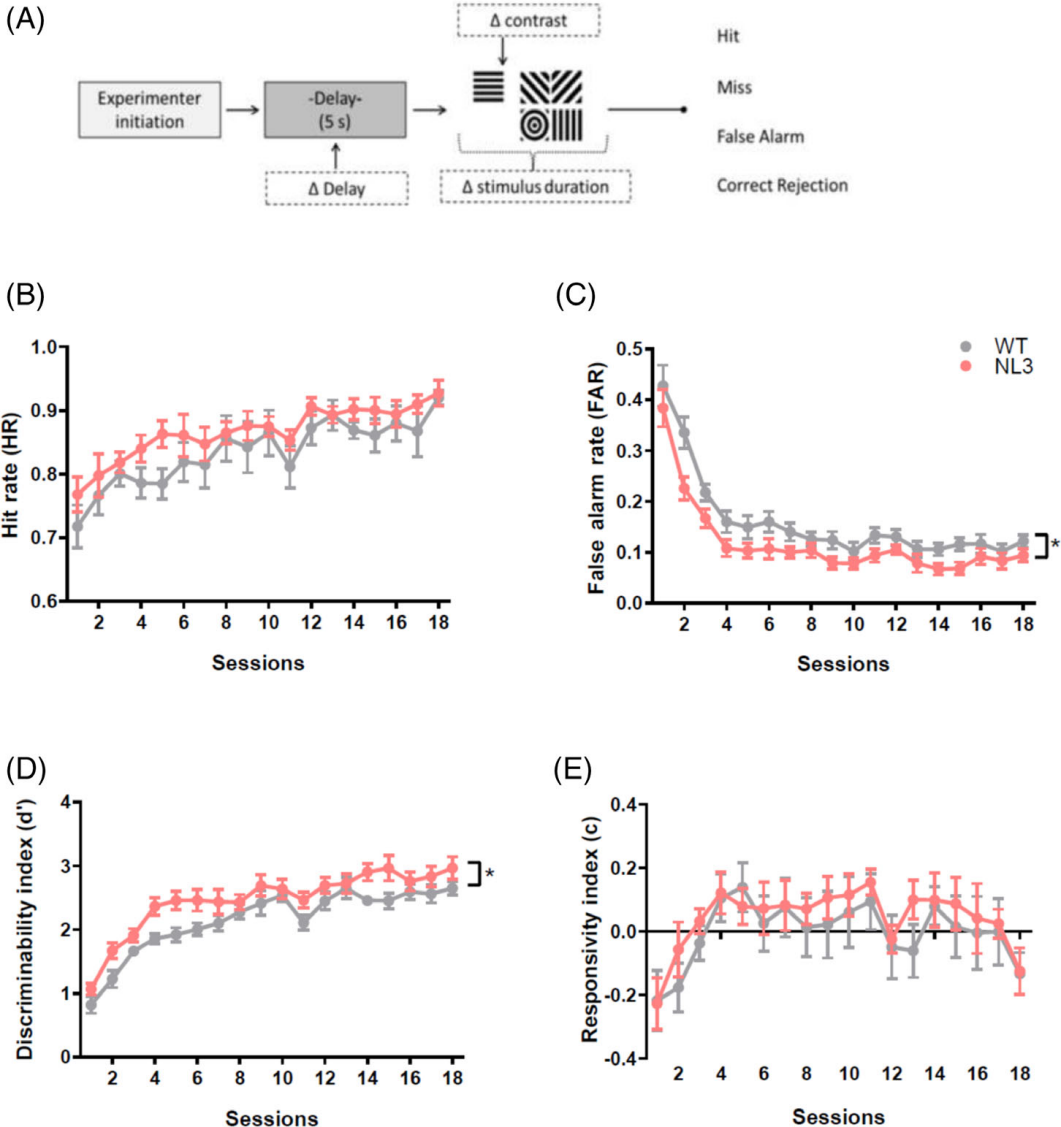
When NL3^R451C^ mice were trained in the rodent continuous‐performance test (rCPT) task, they exhibited improved attentional ability. Schematic of rCPT task (A). No differences in hit rate (HR; B) were seen between WT and NL3^R451C^ mice. NL3^R451C^ mice showed reduced false alarm rates (FAR; C), indicating greater ability to ignore distractor stimuli. NL3^R451C^ mice also showed increased discriminability index (*d*'; D) across training sessions, reflecting improved detection of rewarded image over distractors. No differences were seen in criterion (c; E). Data are presented as mean ± SEM. *p < 0.05

WT and NL3^R451C^ mice acquired early training stages of rCPT similarly (Supplementary Figure 3). During later stages of rCPT training where the difficulty of discrimination was increased by the introduction of four negative distractors (vs. one correct), both WT and NL3^R451C^ mice improved their hit rate (HR) and false alarm rate (FAR) over sessions (Figure 4B; RM ANOVA, *F*_17,340_ = 11.391, *p* < 0.0001; Figure 4C; RM ANOVA, *F*_17,340_ = 17.391, *p* < 0.0001, respectively). The discriminability index (*d*') reflects an animal's ability to distinguish target from non‐target stimuli, and all mice showed improvement in *d*' over sessions (Figure 4D; RM ANOVA, *F*_17,340_ = 46.644, *p* < 0.0001). Response criterion (*c*) describes the animal's propensity to respond to any stimulus and as training progressed, mice adopted a more conservative responses strategy (*c*) as they were less willing to respond to distractor stimuli (Figure 4E; RM ANOVA, *F*_17,340_ = 4.818, *p* < 0.0001). No genotype differences were seen in HR (Figure 4B; ANOVA, *F*_1,20_ = 1.290, *p* = 0.270). NL3 mice made fewer incorrect responses, as reflected by lower FARs (Figure 4C; ANOVA, *F*_1,20_ = 5.686, *p* = 0.027) and this effect was seen equally over all sessions (RM ANOVA, session*geno: *F*_17,340_ = 0.926, *p* = 0.543). NL3 mice responded to stimuli more sensitively, showing consistently higher *d*' across all sessions (Figure 4D; ANOVA, *F*_1,20_ = 5.850, *p* = 0.025; session*geno: *F*_17,340_ = 0.993, *p* = 0.466). No differences in criterion were seen between NL3^R451C^ and WT mice at this stage of training (ANOVA: *F*_1,20_ = 0.284, *p* = 0.600). When the probability of the rewarded image (S+) was reduced (33.3%), due to extensive training at 50% probability, improvement over sessions was no longer observed. Like earlier training sessions, reduced FAR was observed in NL3^R451C^ mice compared to WT littermates (Supplementary Figure 4C; ANOVA, *F*_1,20_ = 10.938 *p* = 0.004).

### 3.3 NL3^R451C^ performance and participation was impaired during attentionally challenging parameters

**FIGURE 5:**
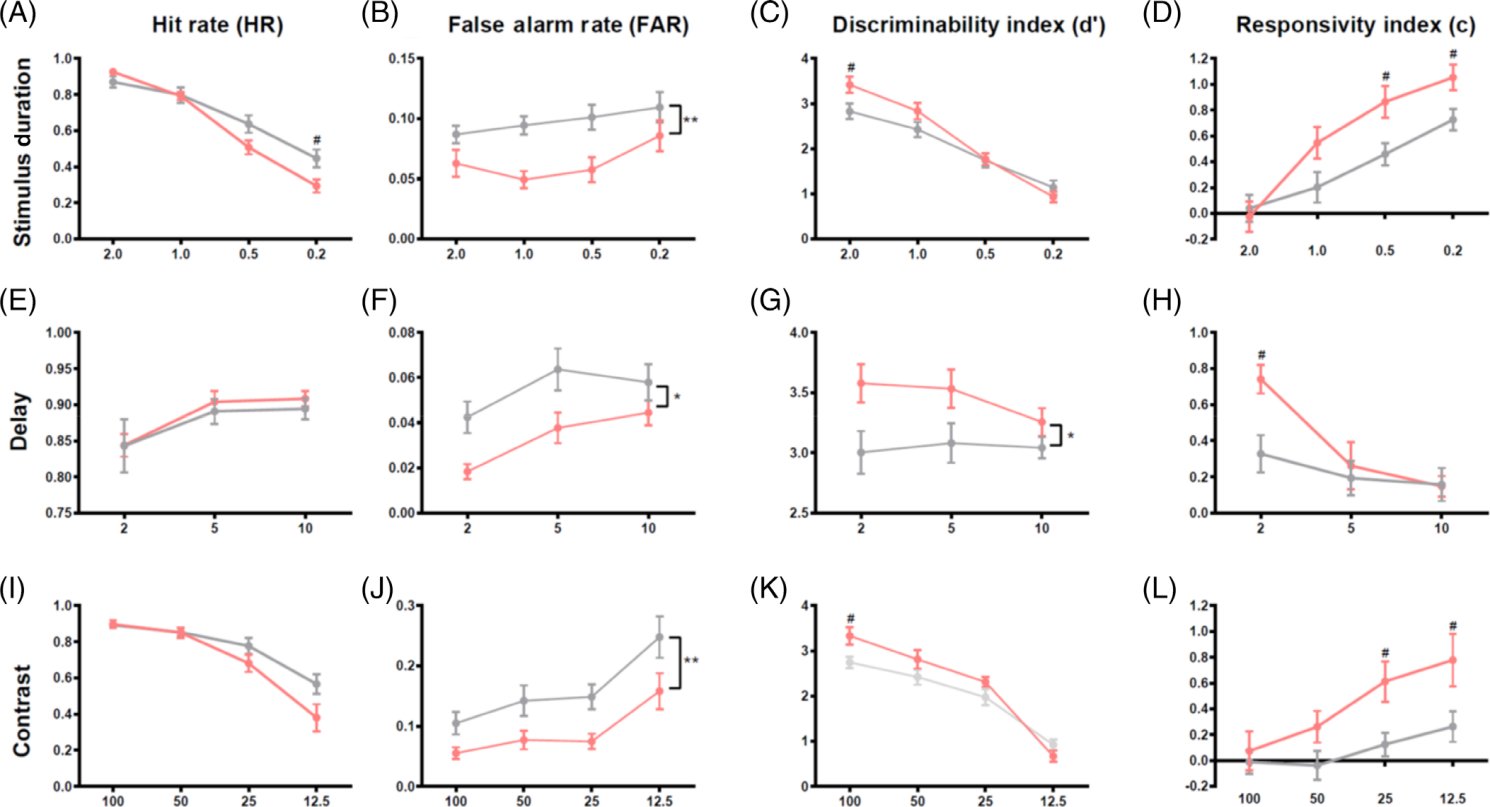
When attention was taxed in the rodent continuous‐performance test (rCPT) by reducing stimulus duration, increasing delay between trials, and reducing the contrast of stimuli, NL3^R451C^ mice consistently exhibited reduced false alarm rates and responded more conservatively. (A–D): In the stimulus duration probe, stimulus duration was reduced from 2.0 to 0.2 s. (E–H): In the delay probe, the time between trials was decreased to 2 and increase d to 10, relative to 5 s baseline. (I–L): In the contrast probe, stimuli were decreased to 12.5% of original brightness. Data are presented as mean ± SEM. *p < 0.05, **p < 0.01

Following acquisition of the rCPT, we investigated how varying task parameters might alter attention in NL3 mice. Reducing stimulus duration (Figure 5A–D) decreased HR (Figure 5A; RM ANOVA, *F*_3,57_ = 249.733, *p* < 0.001) and increased FAR (Figure 5B; RM ANOVA, *F*_3,57_ = 4.310, *p* = 0.008). Consequently, mice responded with less sensitivity to the target, with *d*' decreasing with stimulus duration (Figure 5C; RM ANOVA, *F*_3,57_ = 131.336, *p* < 0.001), and more liberally, with increasing *c* (Figure 5D; RM ANOVA, duration effect, *F*_3,57_ = 90.673, *p* < 0.001). Relative to WT littermates, NL3^R451C^ mice exhibited lower HRs with decreasing stimulus duration (Figure 5A; RM ANOVA, duration*genotype interaction, *F*_3,57_ = 11.208, *p* < 0.0001; pairwise comparison: 0.2 s, *p* = 0.030). Overall, NL3^R451C^ mice made less incorrect responses than WT littermates during the stimulus duration probe (Figure 5B; RM ANOVA, genotype effect, *F*_1,19_ = 9.798, *p* = 0.006), and this did not change with duration (RM ANOVA, duration*genotype interaction, *F*_3,57_ = 1.138, *p* = 0.341). Similar to training performance, NL3^R451C^ mice exhibited higher *d*' at 2 s compared to WT mice, however, this advantage was lost at lesser durations (Figure 5C; RM ANOVA, duration*genotype interaction, *F*_3,57_ = 0.671, *p* = 0.004; pairwise comparison: 2.0 s, *p* = 0.012). NL3^R451C^ mice responded more conservatively to stimuli when stimulus durations were decreased compared to WT mice (Figure 5D; RM ANOVA, duration*genotype interaction, *F*_3,57_ = 7.201, *p* < 0.0001; pairwise comparison: 0.5 s *p* = 0.026, 0.2 s *p* = 0.041).

Manipulating the delay between trials or intertrial interval (ITI) had a varying effect on rodents' ability to attend to stimuli (Figure 5E–H). Regardless of genotype, mice were less likely to respond to stimuli when the intertrial interval was decreased to 2.0 s, making fewer correct (HR; Figure 5E; RM ANOVA, ITI effect, *F*_2,38_ = 10.536, *p* < 0.0001) and incorrect responses (FAR; Figure 5F; ITI effect, *F*_2,38_ = 0.9448, *p* = 0.002). Despite decreases in HR and FAR, no decreases in *d*' were observed with varying ITI (Figure 5G; RM ANOVA, ITI effect, *F*_2,38_ = 0.995, *p* = 0.379). All mice were less willing to respond to stimuli (*c*) when the ITI was reduced (Figure 5H; RM ANOVA, ITI effect, *F*_2,38_ = 18.448, *p* < 0.001). No genotype differences were seen in HR when delays were altered (Figure 5I; RM ANOVA, genotype effect, *F*_1,19_ = 0.142, *p* = 0.710). Regardless of ITI, NL3 mice exhibited lower FARs compared to WT mice (Figure 5F; RM ANOVA, genotype effect, *F*_1,19_ = 7.232, *p* = 0.015). Similarly, ITI did not affect the higher *d*' exhibited by NL3^R451C^ mice (Figure 5G; RM ANOVA, genotype effect, *F*_1,19_ = 7.217, *p* = 0.015). Relative to WT mice, NL3^R451C^ mice responded more conservatively to stimuli (*c*) when the ITI was decreased (Figure 5H; RM ANOVA, genotype*ITI interaction, *F*_2,38_ = 5.706, *p* = 0.007; pairwise comparison: 2.0 s *p* = 0.006).

When stimulus contrast was reduced (Figure 5I–L), mice showed a remarkably similar phenotype to the stimulus duration probe (Figure 5A–D). Both NL3^R451C^ and WT mice made fewer correct responses (HR, Figure 5I; RM ANOVA, contrast effect, *F*_3,54_ = 79.612, *p* < 0.0001) and more incorrect responses (FAR) to stimuli of lower contrasts (Figure 5J; contrast effect, *F*_3,54_ = 17.903, *p* < 0.0001). Consequently, *d*' decreased with decreasing contrast (Figure 5K; RM ANOVA, contrast effect, *F*_3,54_ = 103.878, *p* < 0.0001) and mice responded more liberally (*c*, Figure 5L; RM ANOVA, contrast effect *F*_3,54_ = 18.005, *p* < 0.0001). Compared to WT littermates, NL3^R451C^ mice exhibited lower HRs when image contrast was decreased (Figure 5I; RM ANOVA, contrast*genotype interaction, *F*_3,54_ = 4.501, *p* = 0.007); however, pairwise comparisons were not significant. As with all other rCPT training and probe sessions, NL3^R451C^ mice exhibited a lower FAR compared to WT mice, regardless of contrast (Figure 5J; RM ANOVA, genotype effect, *F*_1,18_ = 9.212, *p* = 0.007). Similar to training conditions, NL3 mice responded to stimuli more sensitively at 100% stimulus contrast compared to WT littermates, however, this effect was lost at lower contrasts (Figure 5K; RM ANOVA, contrast*genotype interaction, *F*_3,54_ = 3.595, *p* = 0.019; pairwise comparison: 100%, *p* = 0.015). NL3^R451C^ mice responded more conservatively (*c*) to stimuli of lower contrasts (Figure 5L; RM ANOVA, contrast*genotype interaction, *F*_3,54_ = 7.201, *p* < 0.0001; pairwise comparison: 25%, *p* = 0.026, 12.5%, *p* = 0.041). When probe performance was scrutinised over time as a measure of sustained attention, NL3^R451C^ mice did not show any differences relative to WT mice (Supplementary Figure 5).

### 3.4 NL3 mice were slower at responding to correct stimuli but faster to collect reward in rCPT

**FIGURE 6:**
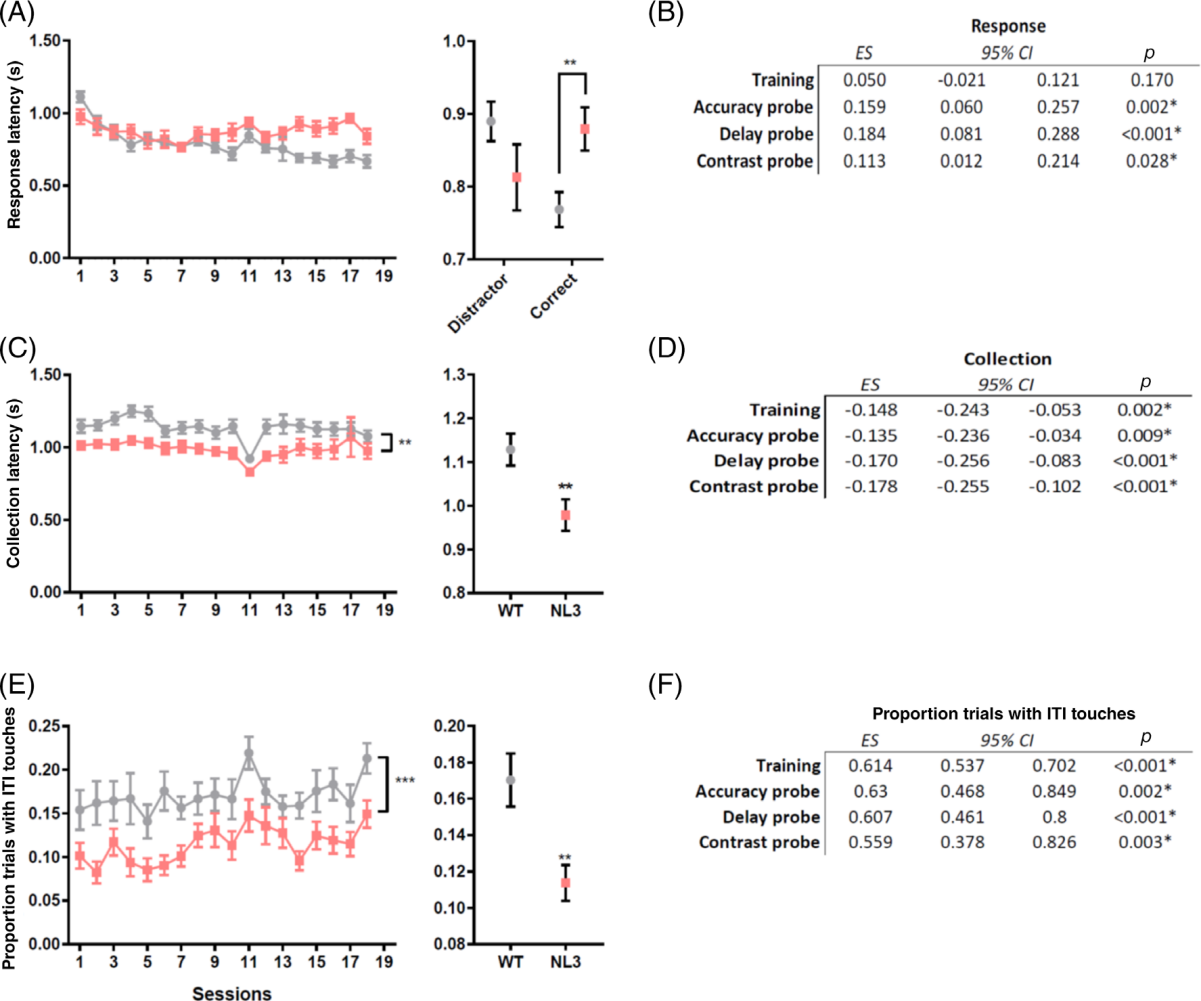
NL3^R451C^ mice displayed slower response latencies, quicker reward latencies and made less touches to the screen in rCPT training and probes. NL3^R451C^ mice were slower to respond to correct stimuli (correct) during training compared to distractor stimuli (distractor) (A). NL3^R451C^ mice also exhibited lower response latencies during all probes (B). Quicker reward collection latencies were seen in NL3^R451C^ mice over the training period (C) and also during all probes (D). NL3^R451C^ mice made less touches to the screen during the inter trial interval (ITI) (E) and during all probes (F). Data are presented as mean ± SEM for visualisation purposes. **p < 0.01

Relative to WT mice, NL3^R451C^ mice were significantly slower to respond to stimuli across all probes (Figure 6B; accuracy: Median regression; Coef. = 0.159; *p* = 0.002; delay: Median regression; Coef. = 0.184; *p* < 0.001; contrast: Median regression; Coef. = 0.113; *p* = 0.028) but not during training (Figure 6A; median regression; Coef. = 0.050; *p* = 0.17). When response times for training were grouped into trials where the correct stimulus was displayed (correct) versus those where distractors were displayed (distractor), NL3^R451C^ mice were only slower when the trial was correct (Figure 6A, interaction of correct stimulus*genotype; median regression; Coef. = 0.211; *p* = 0.009). NL3^R451C^ mice were faster at collecting rewards compared to WT mice during training (Figure 6C; median regression; Coef. = − 0.148; *p* = 0.002) and also all probes (Figure 6D; accuracy: Median regression; Coef. = − 0.135; *p* = 0.009; delay: Median regression; Coef. = − 0.170; *p* < 0.001; contrast: Median regression; Coef. = − 0.178; *p* < 0.001).Relative to WT mice, NL3^R451C^ mice were less likely to prematurely touch the screen at least once during the ITI for training (Figure 6E; logistic regression; OR = 0.614; *p* < 0.001) and during all probes (Figure 6F; accuracy: Logistic regression; OR = 0.631; *p* = 0.002; delay: Logistic regression; OR = 0.608; *p* < 0.001; contrast: Logistic regression; OR = 0.559; *p* = 0.003).

## 4 DISCUSSION

The present study assessed attention in mice expressing the ASD‐associated R451C mutation in NL3 using the 5CSRTT and rodent continuous performance task (rCPT) and revealed a complex phenotype. Attentional performance in the 5CSRTT was largely intact in NL3^R451C^ mice, as reflected by absence of changes in accuracy and omissions in training or when attention was taxed in the probes. This indicates intact capacity for divided attention in this mouse model, specifically in the visuospatial modality. The rCPT is intended to tax vigilance, the capacity to detect rare targets over an extended period. According to signal detection theory, a detector extracts as much information from the stimulus as possible to internally compute their confidence in the correctness of the stimulus. Every presented stimulus is therefore perceived on a continuum: From certainly wrong to certainly right with some ambiguity in between. In contrast to the 5CSRTT (where responding is always beneficial) the introduction of false alarms in the rCPT make responses “risky”, particularly when image discrimination is ambiguous. It is in the context of introducing risk to responding that more observations were made. There were five key findings. First, a reduced false alarm rate was characteristic of NL3^R451C^ mouse performance, irrespective of the CPT probe variations. Second, when the task was less demanding (longer stimulus duration, enhanced stimulus contrast) the NL3^R451C^ mice were better at detecting the target, but this superior performance disappeared when the task became more demanding (shorter stimulus duration, reduced stimulus contrast); instead this led to NL3 mice detecting the target in a similar manner as the WT mice but were more conservative in their bias to respond. Third, in both tasks, NL3^R451C^ mice consistently showed less engagement with the screen during inter‐trial intervals, suggestive of less impulsivity. Together these results suggest that NL3 mice display higher accuracy due in part to their inhibiting responses in the face of uncertainty. Fourth, with any delay between trials the NL3^R451C^ mice detected the targets more readily than the WT mice; with a longer delay between trials they were more likely to respond to the target–the delay benefitted their performance. Fifth, regardless of the task, or level of difficulty, the NL3^R451C^ mice were slower to respond, independent of differences in locomotor activity. The NL3^R451C^ mice therefore showed a cautious but accurate response style, with a bias to conservative responding when the trials were more difficult. Their performance greatly benefitted from more stimulus processing time and pre‐trial preparation time.

When assessed on the rCPT, NL3^R451C^ mice showed enhanced ability to discriminate (*d*') between stimuli during training and at baseline during the probe tests, mainly driven by improved response inhibition (fewer FARs). This advantage was lost; however, when attention was taxed during the probes, with NL3^R451C^ mice showing similar *d*' to WT mice and in some cases, lower hit rates. The increase in difficulty of the task corresponded with NL3^R451C^ mice becoming more conservative in responding to the target, as reflected by higher responsivity indexes (*c*). NL3^R451C^ mice showed adaptability in responding to the changing contingences of the rCPT. Greater adaptability leading to improved performance has been described in previous work assessing motor learning in NL3^R451C^ mice.^34^ In this study, mice were trained to balance on an accelerating rotarod, with the end point being time to fall or the end of trial (300 s). While no differences were seen on the first trial, after training NL3^R451C^ mice showed enhanced ability to remain on the rotarod compared to WT mice, and continued to improve when the task was increased in difficulty. Are there parallels between the mouse CPT results and clinical studies that employ CPT paradigms to assess attention in ASD? Conflicting findings in the human literature make this comparison difficult, nevertheless, some researchers have found that individuals with ASD exhibited poorer focused attention and slower reaction times compared to unaffected siblings and typically developing people.^35^, ^36^, ^37^, ^38^ Garretson^39^ in particular noted that people with ASD exhibited enhanced discriminability but difficulties in sustaining attention throughout the CPT. Other studies have reported intact sustained attention in people with ASD^9^, ^37^, ^40^, ^41^, ^42^, ^43^, ^44^ The lack of consensus on whether sustained attention is altered in ASD was reflected in a recent meta‐analysis examining patterns of cognitive functioning in ASD. This analysis showed that while changes in attention and vigilance were least altered in their large sample, reduced processing speed (or slower response times) was consistently seen.^45^

Slower response times were consistently seen in NL3^R451C^ mice across both tasks in this study. In line with our observations, a recent study assessing NL3^R451C^ mice on a transitive interference touchscreen task also reported longer response latencies.^46^ In our study, NL3^R451C^ mice were slower to respond to the stimuli but made less incorrect responses (fewer false alarms), demonstrating a slower speed‐higher accuracy trade‐off response style. The slower response latencies to stimuli were not a result of impaired motor function, as NL3^R451C^ mice were quicker to retrieve their rewards. Latency to collect reward is regarded as an indication of a rodent's motivation to obtain reward however NL3^R451C^ mice showed no difference in saccharine preference, indicative of equal preference for reward (Supplementary Figure 6). While NL3 mice were quicker to collect their reward, the difference between genotypes was only 200 ms. Given the small size of the apparatus, this effect is likely to be enhanced by floor effects (i.e., mice cannot run any quicker), and thus the biological significance of this effect is questionable. Despite this, in the absence of a test of motivation (e.g., progressive ratio), we cannot determine if reward latencies are reflective of increased motivation in this task. The relationship between motivation and attention is important to consider as motivation can redirect attention to rewarded stimuli.^47^ This constitutes an area worth following up in the NL3^R451C^ mouse. A lack of vigilance decrement was seen in this study, congruent with another using the rCPT.^27^ Other studies utilising aged rats, and task parameters that further taxed attention have described vigilance decrements, adding caution to the translatability of this task used in this study.^48^, ^49^ Differences exist between the rCPT and tasks used to assess attention in humans that could explain the lack of translatability of some measures. The event rate in the rCPT is much higher compared to the Conners CPT 90% signal to 10% noise ratio, making this rodent task more similar to go‐nogo tasks. Furthermore, the presence of reward in the rodent task could help to maintain focus during the session.

NL3^R451C^ mice responded to the rCPT with a cautious but accurate response style, with a bias to conservative responding when the trials were more difficult. NL3^R451C^ mice benefitted in their performance with access to more time – time to process the visual stimuli within the trial and between the trials (ITI). A longer stimulus duration was associated with better stimulus detection (*d*'). Over the three delay period probes, NL3^R451C^ mice also showed better *d*' performance than the WT mice, and with an increase in delay the NL3 mice showed a less conservative responding style. These findings, along with slower response latencies, could be interpreted to suggest that NL3^R451C^ mice have a slower information processing speed than their WT controls. Increased processing times might be a result of subtle differences in sensory processing, as the NL3^R451C^ mutation has been found to cause an increase in inhibitory currents in sensory cortex.^11^, ^12^ Additionally, NL3 has been found to be expressed in retinal astrocytes^50^ and has been suggested to have a protective role. Further interrogations of sensory function in these mice will be required to determine at what stage of the decision making process this delay might occur.

This slow but accurate responding of the NL3^R451C^ mice may have resulted from the extended training to which mice were subjected over time, and thus their responses may be stereotyped. This is in line with evidence from the study characterising motor learning in NL3^R451C^ mice where mice were shown to develop restrictive motor routines over time.^34^ Further work investigating the interaction between the unique NL3^R451C^ mouse response style and time in training is warranted, in particular an assessment of habitual behaviour would be valuable. A decrease in impulsivity (lower FARs and decreased ITI/premature touches) in NL3^R451C^ mice was observed alongside slower response times across both tasks and this could confer more resistance to distractibility. Resistance to distractor interference has been associated with focused attention or selective enhancement for target stimuli^51^ and further interrogation using dedicated flanker‐based attention tasks and the rodent‐modified Stroop task^51^ will be useful for a clearer understanding of the underlying driver of the slower but more accurate response displayed by NL3^R451C^ mice.

In conclusion, this data show that an autism‐linked gene mutation causes differences in response style in attention tasks in mice. Whereas WT mice show a relatively liberal response strategy (at the cost of increased errors), NL3^R451C^ mice showed a different attention phenotype, which manifested as trading down speed for increased signal selectivity. These findings are in line with clinical data suggesting that individuals with ASD possess slower response times, indicative of slower processing speed. The NL3^R451C^ mouse model of ASD will be a useful tool in informing underlying circuitry of attentional abnormalities in ASD and investigating novel therapeutic approaches.

## Supporting information


**Supplementary Figure 1** Timeline of training and probes for both 5‐CSRTT and rCPT.
**Supplementary Figure 2:** Data from 5CSRTT probes. (*) denotes significant (*p* < 0.05) main effect of genotype. (#) denotes significant (*p* < 0.05) main effect of task difficulty. Task difficulty refers to one of stimulus duration, relative stimulus contrast, or longer delays, where appropriate.
**Supplementary Figure 3:** WT and NL3^R451C^ mice acquire basic visual discrimination. Mice showed no difference in hit rate (A), false alarm rate (B), discriminability index (*d*'; C) and criterion (c; D). Data are presented as mean ± SEM
**Supplementary Figure 4**: WT and NL3^R451C^ mice were trained to discriminate S+ from four distractor images at a probability of 33.3%. Mice showed no difference in hit rate (A), discriminability index (*d*'; C) and criterion (c; D), however NL3 mice exhibited lower false alarm rates (B). Data are presented as mean ± SEM
**Supplementary Figure 5:** WT and NL3^R451C^ mice do not show differences in rCPT performance over time. A‐D: Stimulus duration probe. E‐H: Stimulus delay probe. I‐L: Stimulus contrast probe. For each mouse, all sessions were divided into 5 min time bins to calculate performance indicators. Data are presented as mean ± SEM for each group. (*) Denotes a significant main effect of genotype, whereas (#; top right corner) denotes a significant main effect of time. There were no significant interactions of genotype with time. Statistics were calculated using 2‐way repeated measures ANOVAs.
**Supplementary Figure 6:** NL3^R45C1^ mice do not show any difference in saccharine preference, indicating similar inclination for a sweet reward.
**Supplementary Table 1: Detail of regression results for the 5CSRTT grouped by probe.** (**) The effects of interactions were computed in separate models. However, for visualisation purposes are displayed alongside the main effects as independent variables.

## Data Availability

The data that support the findings of this study are available from the corresponding author upon reasonable request.
