# Supplementary material for: Mice with an autism‐associated R451C mutation in neuroligin‐3 show a cautious but accurate response style in touchscreen attention tasks
Source: Genes Brain Behav. 2021 Jul 2;21(1):e12757. doi: 10.1111/gbb.12757 (PMC9744539; doi:10.1111/gbb.12757)
Supplement: Supplementary file 1 — Supplementary Figure 1 Timeline of training and probes for both 5‐CSRTT and rCPT. Supplementary Figure 2: Data from 5CSRTT probes. (*) denotes significant (p < 0.05) main effect of genotype. (#) denotes significant (p < 0.05) main effect of task difficulty. Task difficulty refers to one of stimulus duration, relative stimulus contrast, or longer delays, where appropriate. Supplementary Figure 3: WT and NL3R451C mice acquire basic visual discrimination. Mice showed no difference in hit rate (A), false alarm rate (B), discriminability index (d'; C) and criterion (c; D). Data are presented as mean ± SEM Supplementary Figure 4: WT and NL3R451C mice were trained to discriminate S+ from four distractor images at a probability of 33.3%. Mice showed no difference in hit rate (A), discriminability index (d'; C) and criterion (c; D), however NL3 mice exhibited lower false alarm rates (B). Data are presented as mean ± SEM Supplementary Figure 5: WT and NL3R451C mice do not show differences in rCPT performance over time. A‐D: Stimulus duration probe. E‐H: Stimulus delay probe. I‐L: Stimulus contrast probe. For each mouse, all sessions were divided into 5 min time bins to calculate performance indicators. Data are presented as mean ± SEM for each group. (*) Denotes a significant main effect of genotype, whereas (#; top right corner) denotes a significant main effect of time. There were no significant interactions of genotype with time. Statistics were calculated using 2‐way repeated measures ANOVAs. Supplementary Figure 6: NL3R45C1 mice do not show any difference in saccharine preference, indicating similar inclination for a sweet reward. Supplementary Table 1: Detail of regression results for the 5CSRTT grouped by probe. (**) The effects of interactions were computed in separate models. However, for visualisation purposes are displayed alongside the main effects as independent variables. [file GBB-21-e12757-s001.docx]

**Supplementary Figure 1:** Timeline of training and probes for both 5-CSRTT and rCPT.

**Supplementary Figure 2:** Data from 5CSRTT probes. (*) denotes significant (p < 0.05) main effect of genotype. (#) denotes significant (p < 0.05) main effect of task difficulty. Task difficulty refers to one of stimulus duration, relative stimulus contrast, or longer delays, where appropriate.

**Supplementary Figure 3:** WT and NL3^R451C^ mice acquire basic visual discrimination. Mice showed no difference in hit rate (A), false alarm rate (B), discriminability index (d’; C) and criterion (c; D). Data are presented as mean ± SEM

**Supplementary Figure 4**: WT and NL3^R451C^ mice were trained to discriminate S+ from four distractor images at a probability of 33.3%. Mice showed no difference in hit rate (A), discriminability index (d’; C) and criterion (c; D), however NL3 mice exhibited lower false alarm rates (B). Data are presented as mean ± SEM

**Supplementary Figure 5:** WT and NL3^R451C^ mice do not show differences in rCPT performance over time. A-D: Stimulus duration probe. E-H: Stimulus delay probe. I-L: Stimulus contrast probe. For each mouse, all sessions were divided into 5-minute time bins to calculate performance indicators. Data are presented as mean ± SEM for each group. (*) Denotes a significant main effect of genotype, whereas (#; top right corner) denotes a significant main effect of time. There were no significant interactions of genotype with time. Statistics were calculated using 2-way repeated measures ANOVAs.

**Supplementary Figure 6:** NL3^R45C1^ mice do not show any difference in saccharine preference, indicating similar inclination for a sweet reward.

**Supplementary Table 1: Detail of regression results for the 5CSRTT grouped by probe.** (**) The effects of interactions were computed in separate models. However, for visualisation purposes are displayed alongside the main effects as independent variables.

|  |  | |  | |  | |  | |  | |  | |
| --- | --- | --- | --- | --- | --- | --- | --- | --- | --- | --- | --- | --- |
| Brightness Probe | | | | | | | | | | | |  |
| Initiation Latency (s) | Coef. | Std. Err. | | t | | P>t | | 95% Conf. Interval | | | |  |
| Genotype (NL3^R451C^) | -0.70497 | 0.336075 | | -2.1 | | 0.036 | | -1.36382 | | -0.04612 | |  |
| Successive Day | 0.067 | 0.081389 | | 0.82 | | 0.41 | | -0.09256 | | 0.226557 | |  |
| Brightness (20% increment) | 0.027263 | 0.029378 | | 0.93 | | 0.353 | | -0.03033 | | 0.084857 | |  |
|  |  |  | |  | |  | |  | |  | |  |
| Premature Response (0 or 1) | OR | Std. Err. | | z | | P>z | | 95% Conf. Interval | | | |  |
| Genotype (NL3^R451C^) | 1.076664 | 0.243844 | | 0.33 | | 0.744 | | 0.690716 | | 1.678267 | |  |
| Successive Day | 1.037999 | 0.089444 | | 0.43 | | 0.665 | | 0.876695 | | 1.22898 | |  |
| Brightness (20% increment) | 0.998115 | 0.03957 | | -0.05 | | 0.962 | | 0.923497 | | 1.078763 | |  |
|  |  |  | |  | |  | |  | |  | |  |
| Responded (0 or 1) | OR | Std. Err. | | z | | P>z | | 95% Conf. Interval | | | |  |
| Genotype (NL3^R451C^) | 0.830771 | 0.184395 | | -0.84 | | 0.404 | | 0.537715 | | 1.283544 | |  |
| Successive Day | 1.10683 | 0.052951 | | 2.12 | | 0.034 | | 1.007765 | | 1.215633 | |  |
| Brightness (20% increment) | 1.317934 | 0.061002 | | 5.96 | | 0 | | 1.203635 | | 1.443087 | |  |
| Genotype x Brightness interaction** | 0.935904 | 0.086001 | | -0.72 | | 0.471 | | 0.781654 | | 1.120595 | |  |
|  |  |  | |  | |  | |  | |  | |  |
| Response Latency (s) | Coef. | Std. Err. | | t | | P>t | | 95% Conf. Interval | | | |  |
| Genotype (NL3^R451C^) | 0.22599 | 0.060283 | | 3.75 | | 0 | | 0.107806 | | 0.344175 | |  |
| Successive Day | 0.003006 | 0.014546 | | 0.21 | | 0.836 | | -0.02551 | | 0.031523 | |  |
| Brightness (20% increment) | -0.02799 | 0.009395 | | -2.98 | | 0.003 | | -0.04641 | | -0.00957 | |  |
| Correct Response | -0.50401 | 0.140836 | | -3.58 | | 0 | | -0.78012 | | -0.2279 | |  |
|  |  |  | |  | |  | |  | |  | |  |
| Correct if responded (0 or 1) | OR | Std. Err. | | z | | P>z | | 95% Conf. Interval | | | |  |
| Genotype (NL3^R451C^) | 1.044433 | 0.18451 | | 0.25 | | 0.806 | | 0.738766 | | 1.47657 | |  |
| Successive Day | 0.973808 | 0.048288 | | -0.54 | | 0.592 | | 0.883619 | | 1.073202 | |  |
| Brightness (20% increment) | 1.315703 | 0.064057 | | 5.64 | | 0 | | 1.195957 | | 1.447438 | |  |
| Genotype x Brightness interaction** | 1.166262 | 0.109522 | | 1.64 | | 0.101 | | 0.9702 | | 1.401944 | |  |
|  |  |  | |  | |  | |  | |  | |  |
| Reward Collection Latency (s) | Coef. | Std. Err. | | t | | P>t | | 95% Conf. Interval | | | |  |
| Genotype (NL3^R451C^) | -0.13535 | 0.054769 | | -2.47 | | 0.014 | | -0.24273 | | -0.02797 | |  |
| Successive Day | -0.03964 | 0.010144 | | -3.91 | | 0 | | -0.05953 | | -0.01975 | |  |
| Brightness (20% increment) | 0.00191 | 0.003382 | | 0.56 | | 0.572 | | -0.00472 | | 0.008541 | |  |
|  |  |  | |  | |  | |  | |  | |  |
| Initiation Touches (count) | IRR | Std. Err. | | z | | P>z | | 95% Conf. Interval | | | |  |
| Genotype (NL3^R451C^) | 0.19983 | 0.08056 | | -3.99 | | 0 | | 0.090679 | | 0.44037 | |  |
| Brightness (20% increment) | 0.921858 | 0.119355 | | -0.63 | | 0.53 | | 0.715249 | | 1.188148 | |  |
| Premature Trial | 6.167549 | 3.648631 | | 3.08 | | 0.002 | | 1.934435 | | 19.66396 | |  |
| Omitted Trial | 1.723026 | 0.615559 | | 1.52 | | 0.128 | | 0.855453 | | 3.470464 | |  |
| Incorrect Trial | 1.589075 | 0.547044 | | 1.35 | | 0.179 | | 0.809311 | | 3.120135 | |  |
|  |  |  | |  | |  | |  | |  | |  |
| ITI touches (count) | IRR | Std. Err. | | z | | P>z | | 95% Conf. Interval | | | |  |
| Genotype (NL3^R451C^) | 0.585588 | 0.188996 | | -1.66 | | 0.097 | | 0.311078 | | 1.102337 | |  |
| Brightness (20% increment) | 1.023287 | 0.048669 | | 0.48 | | 0.628 | | 0.932208 | | 1.123265 | |  |
| Premature Trial | 86.66975 | 43.85979 | | 8.82 | | 0 | | 32.14485 | | 233.6811 | |  |
| Omitted Trial | 64.42043 | 24.7193 | | 10.86 | | 0 | | 30.36701 | | 136.6612 | |  |
| Incorrect Trial | 103.6473 | 51.96828 | | 9.26 | | 0 | | 38.79445 | | 276.915 | |  |
|  |  |  | |  | |  | |  | |  | |  |
| Front beam breaks (count) | IRR | Std. Err. | | z | | P>z | | 95% Conf. Interval | | | |  |
| Genotype (NL3^R451C^) | 1.253951 | 0.118183 | | 2.4 | | 0.016 | | 1.042453 | | 1.508359 | |  |
| Brightness (20% increment) | 1.00324 | 0.006381 | | 0.51 | | 0.611 | | 0.990811 | | 1.015825 | |  |
| Premature Trial | 1.190881 | 0.055343 | | 3.76 | | 0 | | 1.087205 | | 1.304443 | |  |
| Omitted Trial | 1.487238 | 0.065432 | | 9.02 | | 0 | | 1.364368 | | 1.621173 | |  |
| Incorrect Trial | 1.402644 | 0.058327 | | 8.14 | | 0 | | 1.292859 | | 1.521751 | |  |
|  |  |  | |  | |  | |  | |  | |  |
| Delay Probe | | | | | | | | | | | |  |
| Initiation Latency (s) | Coef. | Std. Err. | | t | | P>t | | 95% Conf. Interval | | | |  |
| Genotype (NL3^R451C^) | -0.36973 | 0.277559 | | -1.33 | | 0.183 | | -0.9138 | | 0.174339 | |  |
| Successive Day | 0.003835 | 0.036643 | | 0.1 | | 0.917 | | -0.06799 | | 0.075662 | |  |
| Long Stimulus Delay | 0.400808 | 0.136278 | | 2.94 | | 0.003 | | 0.133675 | | 0.66794 | |  |
|  |  |  | |  | |  | |  | |  | |  |
| Premature Response (0 or 1) | OR | Std. Err. | | z | | P>z | | 95% Conf. Interval | | | |  |
| Genotype (NL3^R451C^) | 0.9723 | 0.244438 | | -0.11 | | 0.911 | | 0.594028 | | 1.591452 | |  |
| Successive Day | 1.071252 | 0.040181 | | 1.84 | | 0.067 | | 0.995324 | | 1.152972 | |  |
| Long Stimulus Delay | 11.84881 | 2.52574 | | 11.6 | | 0 | | 7.802419 | | 17.99369 | |  |
|  |  |  | |  | |  | |  | |  | |  |
| Responded (0 or 1) | OR | Std. Err. | | z | | P>z | | 95% Conf. Interval | | | |  |
| Genotype (NL3^R451C^) | 0.839396 | 0.149882 | | -0.98 | | 0.327 | | 0.59153 | | 1.191124 | |  |
| Successive Day | 1.024801 | 0.026552 | | 0.95 | | 0.344 | | 0.974061 | | 1.078185 | |  |
| Long Stimulus Delay | 1.417196 | 0.232354 | | 2.13 | | 0.033 | | 1.027716 | | 1.954281 | |  |
| Genotype x Delay interaction** | 1.563932 | 0.492256 | | 1.42 | | 0.155 | | 0.843913 | | 2.898265 | |  |
|  |  |  | |  | |  | |  | |  | |  |
| Response Latency (s) | Coef. | Std. Err. | | t | | P>t | | 95% Conf. Interval | | | |  |
| Genotype (NL3^R451C^) | 0.151969 | 0.05313 | | 2.86 | | 0.004 | | 0.047822 | | 0.256116 | |  |
| Successive Day | 0.003004 | 0.005399 | | 0.56 | | 0.578 | | -0.00758 | | 0.013586 | |  |
| Long Stimulus Delay | 0.008985 | 0.028754 | | 0.31 | | 0.755 | | -0.04738 | | 0.065349 | |  |
| Correct Response | -0.27899 | 0.084289 | | -3.31 | | 0.001 | | -0.44422 | | -0.11377 | |  |
|  |  |  | |  | |  | |  | |  | |  |
| Correct if responded (0 or 1) | OR | Std. Err. | | z | | P>z | | 95% Conf. Interval | | | |  |
| Genotype (NL3^R451C^) | 1.288983 | 0.242693 | | 1.35 | | 0.178 | | 0.891211 | | 1.864291 | |  |
| Successive Day | 0.944118 | 0.031807 | | -1.71 | | 0.088 | | 0.883791 | | 1.008562 | |  |
| Long Stimulus Delay | 0.640201 | 0.052904 | | -5.4 | | 0 | | 0.544473 | | 0.752759 | |  |
| Genotype x Delay interaction** | 0.943796 | 0.153723 | | -0.36 | | 0.722 | | 0.685863 | | 1.298731 | |  |
|  |  |  | |  | |  | |  | |  | |  |
| Reward Collection Latency (s) | Coef. | Std. Err. | | t | | P>t | | 95% Conf. Interval | | | |  |
| Genotype (NL3^R451C^) | -0.186 | 0.046777 | | -3.98 | | 0 | | -0.27769 | | -0.0943 | |  |
| Successive Day | -0.01699 | 0.005167 | | -3.29 | | 0.001 | | -0.02712 | | -0.00687 | |  |
| Long Stimulus Delay | 0.02599 | 0.02497 | | 1.04 | | 0.298 | | -0.02296 | | 0.074938 | |  |
|  |  |  | |  | |  | |  | |  | |  |
| Initiation Touches (count) | IRR | Std. Err. | | z | | P>z | | 95% Conf. Interval | | | |  |
| Genotype (NL3^R451C^) | 0.375459 | 0.163572 | | -2.25 | | 0.025 | | 0.159856 | | 0.881855 | |  |
| Long Stimulus Delay | 2.4496 | 0.745065 | | 2.95 | | 0.003 | | 1.349571 | | 4.446259 | |  |
| Premature Trial | 2.969053 | 1.070726 | | 3.02 | | 0.003 | | 1.464367 | | 6.019852 | |  |
| Omitted Trial | 6.587205 | 3.119824 | | 3.98 | | 0 | | 2.603492 | | 16.66657 | |  |
| Incorrect Trial | 1.656081 | 0.731036 | | 1.14 | | 0.253 | | 0.697171 | | 3.933901 | |  |
|  |  |  | |  | |  | |  | |  | |  |
| ITI touches (count) | IRR | Std. Err. | | z | | P>z | | 95% Conf. Interval | | | |  |
| Genotype (NL3^R451C^) | 0.640483 | 0.171913 | | -1.66 | | 0.097 | | 0.378474 | | 1.083877 | |  |
| Long Stimulus Delay | 0.986333 | 0.165134 | | -0.08 | | 0.934 | | 0.710418 | | 1.369411 | |  |
| Premature Trial | 104.8975 | 37.24399 | | 13.11 | | 0 | | 52.30519 | | 210.3707 | |  |
| Omitted Trial | 84.96203 | 30.16756 | | 12.51 | | 0 | | 42.3631 | | 170.397 | |  |
| Incorrect Trial | 96.18484 | 36.72125 | | 11.96 | | 0 | | 45.51313 | | 203.2715 | |  |
|  |  |  | |  | |  | |  | |  | |  |
| Front beam breaks (count) | IRR | Std. Err. | | z | | P>z | | 95% Conf. Interval | | | |  |
| Genotype (NL3^R451C^) | 1.349778 | 0.190494 | | 2.13 | | 0.034 | | 1.023604 | | 1.779887 | |  |
| Long Stimulus Delay | 1.23651 | 0.069707 | | 3.77 | | 0 | | 1.107165 | | 1.380966 | |  |
| Premature Trial | 1.200295 | 0.07756 | | 2.83 | | 0.005 | | 1.057512 | | 1.362356 | |  |
| Omitted Trial | 1.693562 | 0.074734 | | 11.94 | | 0 | | 1.553241 | | 1.84656 | |  |
| Incorrect Trial | 1.381455 | 0.077803 | | 5.74 | | 0 | | 1.237079 | | 1.542681 | |  |
|  |  |  | |  | |  | |  | |  | |  |
| Accuracy Probe | | | | | | | | | | | |  |
| Initiation Latency (s) | Coef. | Std. Err. | | t | | P>t | | 95% Conf. Interval | | | |  |
| Genotype (NL3^R451C^) | -1.00174 | 0.489473 | | -2.05 | | 0.041 | | -1.96118 | | -0.0423 | |  |
| Successive Day | 0.072451 | 0.038089 | | 1.9 | | 0.057 | | -0.00221 | | 0.147111 | |  |
| Stimulus Duration (s) | -2.39149 | 0.331175 | | -7.22 | | 0 | | -3.04064 | | -1.74234 | |  |
|  |  |  | |  | |  | |  | |  | |  |
| Premature Response (0 or 1) | OR | Std. Err. | | z | | P>z | | 95% Conf. Interval | | | |  |
| Genotype (NL3^R451C^) | 1.133458 | 0.243473 | | 0.58 | | 0.56 | | 0.743982 | | 1.726824 | |  |
| Successive Day | 0.983808 | 0.01309 | | -1.23 | | 0.22 | | 0.958484 | | 1.009801 | |  |
| Stimulus Duration (s) | 0.73893 | 0.116831 | | -1.91 | | 0.056 | | 0.542027 | | 1.007361 | |  |
|  |  |  | |  | |  | |  | |  | |  |
| Responded (0 or 1) | OR | Std. Err. | | z | | P>z | | 95% Conf. Interval | | | |  |
| Genotype (NL3^R451C^) | 0.770079 | 0.122526 | | -1.64 | | 0.101 | | 0.563771 | | 1.051885 | |  |
| Successive Day | 1.043966 | 0.0153 | | 2.94 | | 0.003 | | 1.014406 | | 1.074388 | |  |
| Stimulus Duration (s) | 8.301998 | 1.102294 | | 15.94 | | 0 | | 6.399775 | | 10.76962 | |  |
| Genotype x Duration interaction** | 0.981458 | 0.26096 | | -0.07 | | 0.944 | | 0.582835 | | 1.652715 | |  |
|  |  |  | |  | |  | |  | |  | |  |
| Response Latency (s) | Coef. | Std. Err. | | t | | P>t | | 95% Conf. Interval | | | |  |
| Genotype (NL3^R451C^) | 0.128798 | 0.093217 | | 1.38 | | 0.167 | | -0.05393 | | 0.311531 | |  |
| Successive Day | -0.00414 | 0.006183 | | -0.67 | | 0.503 | | -0.01626 | | 0.007979 | |  |
| Stimulus Duration (s) | 0.073336 | 0.051993 | | 1.41 | | 0.158 | | -0.02858 | | 0.175257 | |  |
| Correct Response | -1.11804 | 0.070067 | | -15.96 | | 0 | | -1.25539 | | -0.98069 | |  |
|  |  |  | |  | |  | |  | |  | |  |
| Correct if responded (0 or 1) | OR | Std. Err. | | z | | P>z | | 95% Conf. Interval | | | |  |
| Genotype (NL3^R451C^) | 1.141119 | 0.137697 | | 1.09 | | 0.274 | | 0.900777 | | 1.445587 | |  |
| Successive Day | 1.028558 | 0.014608 | | 1.98 | | 0.047 | | 1.000322 | | 1.057592 | |  |
| Stimulus Duration (s) | 12.50292 | 2.304025 | | 13.71 | | 0 | | 8.712707 | | 17.94197 | |  |
| Genotype x Duration interaction** | 0.847896 | 0.328254 | | -0.43 | | 0.67 | | 0.397018 | | 1.810823 | |  |
|  |  |  | |  | |  | |  | |  | |  |
| Reward Collection Latency (s) | Coef. | Std. Err. | | t | | P>t | | 95% Conf. Interval | | | |  |
| Genotype (NL3^R451C^) | -0.09026 | 0.042502 | | -2.12 | | 0.034 | | -0.17358 | | -0.00694 | |  |
| Successive Day | 0.003006 | 0.005204 | | 0.58 | | 0.564 | | -0.0072 | | 0.013209 | |  |
| Stimulus Duration (s) | -0.00628 | 0.033988 | | -0.18 | | 0.854 | | -0.07291 | | 0.060356 | |  |
|  |  |  | |  | |  | |  | |  | |  |
| ITI touches (count) | IRR | Std. Err. | | z | | P>z | | 95% Conf. Interval | | | |  |
| Genotype (NL3^R451C^) | 0.737719 | 0.140299 | | -1.6 | | 0.11 | | 0.508171 | | 1.070956 | |  |
| Successive Day | 0.960403 | 0.124099 | | -0.31 | | 0.755 | | 0.74553 | | 1.237207 | |  |
| Premature Trial | 80.17221 | 38.41309 | | 9.15 | | 0 | | 31.34627 | | 205.051 | |  |
| Omitted Trial | 63.36914 | 32.53234 | | 8.08 | | 0 | | 23.16804 | | 173.327 | |  |
| Incorrect Trial | 78.97749 | 39.1267 | | 8.82 | | 0 | | 29.90918 | | 208.5461 | |  |
|  |  |  | |  | |  | |  | |  | |  |
| Initiation Touches (count) | IRR | Std. Err. | | z | | P>z | | 95% Conf. Interval | | | |  |
| Genotype (NL3^R451C^) | 0.374246 | 0.136895 | | -2.69 | | 0.007 | | 0.182725 | | 0.766508 | |  |
| Successive Day | 0.45785 | 0.089361 | | -4 | | 0 | | 0.312313 | | 0.671207 | |  |
| Premature Trial | 1.638073 | 0.220881 | | 3.66 | | 0 | | 1.257637 | | 2.13359 | |  |
| Omitted Trial | 1.607053 | 0.17469 | | 4.36 | | 0 | | 1.298683 | | 1.988646 | |  |
| Incorrect Trial | 1.080937 | 0.121367 | | 0.69 | | 0.488 | | 0.867418 | | 1.347015 | |  |
|  |  |  | |  | |  | |  | |  | |  |
| Front beam breaks (count) | IRR | Std. Err. | | z | | P>\|z\| | | [95% Conf. Interval] | | | |  |
| Genotype (NL3^R451C^) | 1.38955 | 0.012474 | | 36.65 | | 0 | | 1.365316 | | 1.414215 | |  |
| Successive Day | 1.008292 | 0.007362 | | 1.13 | | 0.258 | | 0.993965 | | 1.022825 | |  |
| Premature Trial | 1.136111 | 0.047531 | | 3.05 | | 0.002 | | 1.04667 | | 1.233195 | |  |
| Omitted Trial | 1.475439 | 0.052348 | | 10.96 | | 0 | | 1.376324 | | 1.581691 | |  |
| Incorrect Trial | 1.278576 | 0.037316 | | 8.42 | | 0 | | 1.207491 | | 1.353846 | |  |
|  |  |  | |  | |  | |  | |  | |  |
| Training | | | | | | | | | | | |  |
| Initiation Latency (s) | Coef. | Std. Err. | | t | | P>t | | 95% Conf. Interval | | | |  |
| Genotype (NL3^R451C^) | -1.48596 | 0.628239 | | -2.37 | | 0.018 | | -2.71736 | | -0.25457 | |  |
| Successive Day | -0.09625 | 0.046084 | | -2.09 | | 0.037 | | -0.18658 | | -0.00592 | |  |
| Stimulus Duration = 16s | 0.483765 | 0.397693 | | 1.22 | | 0.224 | | -0.29574 | | 1.263273 | |  |
| Stimulus Duration = 8s | -0.54967 | 0.470165 | | -1.17 | | 0.242 | | -1.47123 | | 0.371891 | |  |
| Stimulus Duration = 4s | -0.76666 | 0.489124 | | -1.57 | | 0.117 | | -1.72538 | | 0.192057 | |  |
| Stimulus Duration = 2s | -0.92496 | 0.550929 | | -1.68 | | 0.093 | | -2.00482 | | 0.154905 | |  |
|  |  |  | |  | |  | |  | |  | |  |
| Premature Response (0 or 1) | OR | Std. Err. | | z | | P>z | | 95% Conf. Interval | | | |  |
| Genotype (NL3^R451C^) | 0.706809 | 0.102236 | | -2.4 | | 0.016 | | 0.532329 | | 0.938476 | |  |
| Successive Day | 0.894983 | 0.021808 | | -4.55 | | 0 | | 0.853245 | | 0.938764 | |  |
| Stimulus Duration = 16s | 0.435819 | 0.039591 | | -9.14 | | 0 | | 0.364738 | | 0.520753 | |  |
| Stimulus Duration = 8s | 0.359774 | 0.059159 | | -6.22 | | 0 | | 0.260653 | | 0.496589 | |  |
| Stimulus Duration = 4s | 0.446735 | 0.09144 | | -3.94 | | 0 | | 0.299104 | | 0.667233 | |  |
| Stimulus Duration = 2s | 0.595466 | 0.119605 | | -2.58 | | 0.01 | | 0.401685 | | 0.882731 | |  |
|  |  |  | |  | |  | |  | |  | |  |
| Responded (0 or 1) | OR | Std. Err. | | z | | P>z | | 95% Conf. Interval | | | |  |
| Genotype (NL3^R451C^) | 0.754161 | 0.197805 | | -1.08 | | 0.282 | | 0.451031 | | 1.26102 | |  |
| Successive Day | 1.20497 | 0.036716 | | 6.12 | | 0 | | 1.135115 | | 1.279124 | |  |
| Stimulus Duration = 16s | 0.961017 | 0.485495 | | -0.08 | | 0.937 | | 0.357038 | | 2.586712 | |  |
| Stimulus Duration = 8s | 0.407767 | 0.177015 | | -2.07 | | 0.039 | | 0.17414 | | 0.954829 | |  |
| Stimulus Duration = 4s | 0.045252 | 0.017335 | | -8.08 | | 0 | | 0.021358 | | 0.095875 | |  |
| Stimulus Duration = 2s | 0.005654 | 0.002699 | | -10.84 | | 0 | | 0.002218 | | 0.014412 | |  |
| Genotype x Day interaction** | 1.055133 | 0.030476 | | 1.86 | | 0.063 | | 0.997061 | | 1.116587 | |  |
|  |  |  | |  | |  | |  | |  | |  |
| Response Latency (s) | Coef. | Std. Err. | | t | | P>t | | 95% Conf. Interval | | | |  |
| Genotype (NL3^R451C^) | 0.172076 | 0.065309 | | 2.63 | | 0.008 | | 0.044062 | | 0.300089 | |  |
| Successive Day | -0.00786 | 0.005684 | | -1.38 | | 0.167 | | -0.019 | | 0.003285 | |  |
| Stimulus Duration = 16s | -0.16671 | 0.086641 | | -1.92 | | 0.054 | | -0.33654 | | 0.003113 | |  |
| Stimulus Duration = 8s | -0.35727 | 0.074492 | | -4.8 | | 0 | | -0.50329 | | -0.21126 | |  |
| Stimulus Duration = 4s | -0.50835 | 0.086134 | | -5.9 | | 0 | | -0.67719 | | -0.33952 | |  |
| Stimulus Duration = 2s | -0.74569 | 0.073721 | | -10.12 | | 0 | | -0.89019 | | -0.60119 | |  |
| Correct Response | 0.125279 | 0.075631 | | 1.66 | | 0.098 | | -0.02297 | | 0.273524 | |  |
|  |  |  | |  | |  | |  | |  | |  |
| Correct if responded (0 or 1) | OR | Std. Err. | | z | | P>z | | 95% Conf. Interval | | | |  |
| Genotype (NL3^R451C^) | 1.408772 | 0.257261 | | 1.88 | | 0.061 | | 0.984917 | | 2.01503 | |  |
| Successive Day | 1.044245 | 0.018419 | | 2.45 | | 0.014 | | 1.00876 | | 1.080977 | |  |
| Stimulus Duration = 16s | 1.79925 | 0.207004 | | 5.11 | | 0 | | 1.43602 | | 2.254356 | |  |
| Stimulus Duration = 8s | 1.872945 | 0.326268 | | 3.6 | | 0 | | 1.331206 | | 2.635146 | |  |
| Stimulus Duration = 4s | 1.612226 | 0.292561 | | 2.63 | | 0.008 | | 1.129701 | | 2.300852 | |  |
| Stimulus Duration = 2s | 0.985744 | 0.234104 | | -0.06 | | 0.952 | | 0.618888 | | 1.570058 | |  |
| Genotype x Day interaction** | 1.039403 | 0.024842 | | 1.62 | | 0.106 | | 0.991836 | | 1.089252 | |  |
|  |  |  | |  | |  | |  | |  | |  |
| Reward Collection Latency (s) | Coef. | Std. Err. | | t | | P>t | | 95% Conf. Interval | | | |  |
| Genotype (NL3^R451C^) | -0.08631 | 0.044247 | | -1.95 | | 0.051 | | -0.17304 | | 0.000416 | |  |
| Successive Day | 0.01034 | 0.007288 | | 1.42 | | 0.156 | | -0.00394 | | 0.024626 | |  |
| Stimulus Duration = 16s | -0.08644 | 0.022041 | | -3.92 | | 0 | | -0.12964 | | -0.04323 | |  |
| Stimulus Duration = 8s | -0.13205 | 0.037401 | | -3.53 | | 0 | | -0.20536 | | -0.05874 | |  |
| Stimulus Duration = 4s | -0.18536 | 0.052642 | | -3.52 | | 0 | | -0.28854 | | -0.08217 | |  |
| Stimulus Duration = 2s | -0.20981 | 0.077702 | | -2.7 | | 0.007 | | -0.36212 | | -0.05751 | |  |
|  |  |  | |  | |  | |  | |  | |  |
| Initiation Touches (count) | IRR | Std. Err. | | z | | P>z | | 95% Conf. Interval | | | |  |
| Genotype (NL3^R451C^) | 0.431588 | 0.02023 | | -17.93 | | 0 | | 0.393705 | | 0.473117 | |  |
| Successive Day | 0.969874 | 0.017415 | | -1.7 | | 0.088 | | 0.936336 | | 1.004614 | |  |
| Stimulus Duration = 16s | 0.896677 | 0.126599 | | -0.77 | | 0.44 | | 0.67992 | | 1.182537 | |  |
| Stimulus Duration = 8s | 0.651919 | 0.13478 | | -2.07 | | 0.039 | | 0.434723 | | 0.97763 | |  |
| Stimulus Duration = 4s | 0.580277 | 0.166293 | | -1.9 | | 0.058 | | 0.330904 | | 1.01758 | |  |
| Stimulus Duration = 2s | 0.857323 | 0.227233 | | -0.58 | | 0.561 | | 0.509958 | | 1.441301 | |  |
| Premature Trial | 1.061951 | 0.110719 | | 0.58 | | 0.564 | | 0.865681 | | 1.302719 | |  |
| Omitted Trial | 4.914134 | 2.15011 | | 3.64 | | 0 | | 2.084566 | | 11.58452 | |  |
| Incorrect Trial | 1.166751 | 0.120076 | | 1.5 | | 0.134 | | 0.953623 | | 1.427512 | |  |
|  |  |  | |  | |  | |  | |  | |  |
| ITI touches (count) | IRR | Std. Err. | | z | | P>z | | 95% Conf. Interval | | | |  |
| Genotype (NL3^R451C^) | 0.685421 | 0.121768 | | -2.13 | | 0.033 | | 0.48388 | | 0.970907 | |  |
| Successive Day | 0.94041 | 0.017429 | | -3.32 | | 0.001 | | 0.906863 | | 0.975197 | |  |
| Stimulus Duration = 16s | 0.604538 | 0.062768 | | -4.85 | | 0 | | 0.493225 | | 0.740973 | |  |
| Stimulus Duration = 8s | 0.615935 | 0.11397 | | -2.62 | | 0.009 | | 0.428581 | | 0.885192 | |  |
| Stimulus Duration = 4s | 0.572924 | 0.097451 | | -3.27 | | 0.001 | | 0.410499 | | 0.799616 | |  |
| Stimulus Duration = 2s | 0.926794 | 0.165826 | | -0.42 | | 0.671 | | 0.652653 | | 1.316085 | |  |
| Premature Trial | 20.98417 | 6.824757 | | 9.36 | | 0 | | 11.09308 | | 39.69457 | |  |
| Omitted Trial | 14.11103 | 3.658705 | | 10.21 | | 0 | | 8.489055 | | 23.45622 | |  |
| Incorrect Trial | 16.89875 | 5.755937 | | 8.3 | | 0 | | 8.668103 | | 32.94466 | |  |
|  |  |  | |  | |  | |  | |  | |  |
| Front beam breaks (count) | IRR | Std. Err. | | z | | P>z | | 95% Conf. Interval | | | |  |
| Genotype (NL3^R451C^) | 1.329961 | 0.172701 | | 2.2 | | 0.028 | | 1.031115 | | 1.715421 | |  |
| Successive Day | 1.010473 | 0.006236 | | 1.69 | | 0.091 | | 0.998323 | | 1.02277 | |  |
| Stimulus Duration = 16s | 1.008359 | 0.03681 | | 0.23 | | 0.82 | | 0.938733 | | 1.08315 | |  |
| Stimulus Duration = 8s | 0.950231 | 0.044394 | | -1.09 | | 0.275 | | 0.867085 | | 1.04135 | |  |
| Stimulus Duration = 4s | 0.902548 | 0.060815 | | -1.52 | | 0.128 | | 0.790888 | | 1.029972 | |  |
| Stimulus Duration = 2s | 0.86558 | 0.073504 | | -1.7 | | 0.089 | | 0.732865 | | 1.022329 | |  |
| Premature Trial | 0.955432 | 0.023166 | | -1.88 | | 0.06 | | 0.911089 | | 1.001934 | |  |
| Omitted Trial | 1.394594 | 0.048386 | | 9.59 | | 0 | | 1.302911 | | 1.492729 | |  |
| Incorrect Trial | 1.196866 | 0.031008 | | 6.94 | | 0 | | 1.137608 | | 1.25921 | |  |
